# Supplementary material for: Pan-cancer spatial characterization of key immune biomarkers in the tumor microenvironment
Source: Cell Rep Med. 2025 Oct 14;7(10):102418. doi: 10.1016/j.xcrm.2025.102418 (PMC13710152; doi:10.1016/j.xcrm.2025.102418)
Supplement: Supplementary file 1 [file mmc1.pdf]

Cell Reports Medicine, Volume *7*

Supplemental information

Pan-cancer spatial characterization of key immune biomarkers in the tumor microenvironment

James R. Lindsay, Jennifer Altreuter, Joao V. Alessi, Jason L. Weirather, Anita Giobbie-Hurder, Ian Dryg, Katharina Hoebel, Bijaya Sharma, Kristen Felt, F. Stephen Hodi, Neal Lindeman, Lynette M. Sholl, Ethan Cerami, Jonathan A. Nowak, Mark M. Awad, Scott J. Rodig, and William Lotter

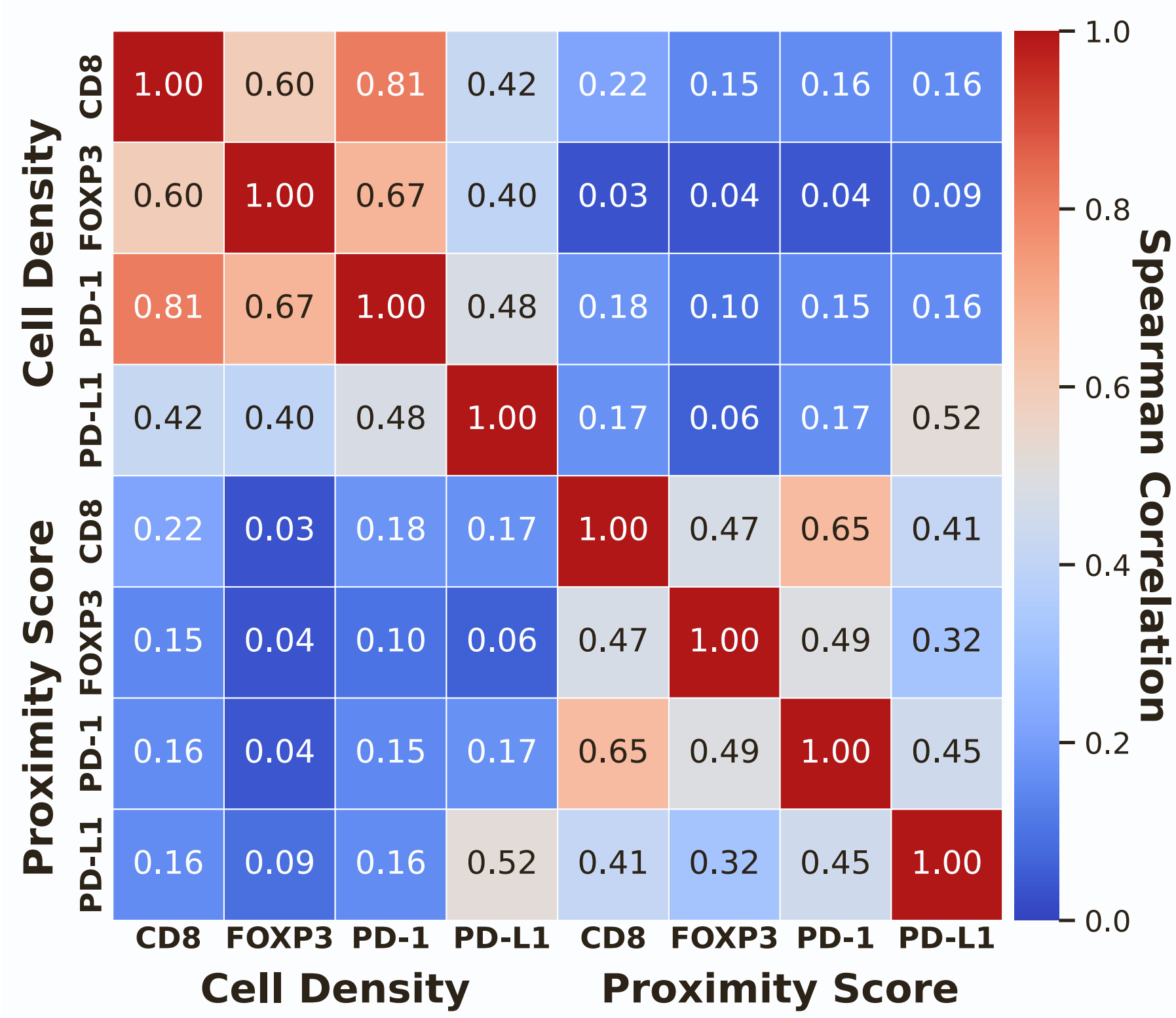

Figure S1. Correlations between the cell density and proximity score metrics across biomarkers. Related to Figure 2. Values represent the Spearman correlation computed across the cohort. Low correlations are generally observed between the cell density and proximity score metrics for the same biomarker, and moderate to high correlations are observed between different biomarkers for the same metric.

		Cell Density				Tumor	Proximity Score					
Factor	Subset	CD8	FOXP3	PD-L1	PD-1	TPS	CD8	FOXP3	PD-L1	PD-1	Variance	PC Rank
Immune Enrichment	All	0.41	0.36	0.44	0.42	0.39	0.22	0.16	0.27	0.21	43%	1
	NSCLC	0.38	0.34	0.43	0.38	0.35	0.27	0.17	0.32	0.28	40%	1
	BRCA	0.42	0.45	0.46	0.45	0.39	0.06	0.13	0.16	0.11	43%	1
	CRC	0.41	0.33	0.42	0.42	0.38	0.27	0.16	0.24	0.25	43%	1
	Other Cancer	0.41	0.35	0.44	0.43	0.39	0.21	0.15	0.27	0.20	43%	1
	Low Stage	0.41	0.37	0.44	0.42	0.40	0.18	0.14	0.27	0.19	43%	1
	High Stage	0.40	0.34	0.43	0.42	0.39	0.24	0.17	0.27	0.22	43%	1
	Genomics	0.40	0.35	0.43	0.41	0.38	0.26	0.17	0.27	0.22	43%	1
Immune-Tumor Mixing	All	-0.18	-0.28	-0.20	-0.22	-0.05	0.49	0.44	0.33	0.50	24%	2
	NSCLC	-0.25	-0.32	-0.19	-0.30	-0.05	0.47	0.45	0.25	0.47	22%	2
	BRCA	-0.10	-0.11	-0.10	-0.14	-0.01	0.57	0.51	0.35	0.49	23%	2
	CRC	-0.20	-0.31	-0.22	-0.22	-0.05	0.39	0.48	0.37	0.49	23%	2
	Other Cancer	-0.16	-0.26	-0.21	-0.20	-0.06	0.50	0.43	0.35	0.51	24%	2
	Low Stage	-0.15	-0.27	-0.18	-0.20	-0.06	0.51	0.44	0.33	0.51	23%	2
	High Stage	-0.19	-0.30	-0.21	-0.23	-0.06	0.47	0.44	0.32	0.50	24%	2
	Genomics	-0.21	-0.27	-0.22	-0.24	-0.06	0.46	0.45	0.33	0.50	23%	2
Immune Evasion	All	-0.33	-0.21	0.21	-0.28	0.54	-0.20	-0.28	0.54	-0.13	11%	3
	NSCLC	-0.33	-0.18	0.24	-0.32	0.55	-0.20	-0.30	0.50	-0.13	17%	3
	BRCA	-0.35	-0.19	0.14	-0.17	0.50	-0.21	-0.31	0.63	0.00	11%	3
	CRC	-0.21	-0.30	0.24	-0.20	0.56	-0.40	-0.07	0.50	-0.19	11%	3
	Other Cancer	-0.37	-0.22	0.22	-0.29	0.57	-0.20	-0.21	0.51	-0.15	11%	3
	Low Stage	-0.31	-0.23	0.19	-0.31	0.52	-0.18	-0.28	0.57	-0.14	11%	3
	High Stage	-0.34	-0.22	0.22	-0.27	0.55	-0.20	-0.27	0.53	-0.14	12%	3
	Genomics	-0.31	-0.22	0.21	-0.27	0.56	-0.22	-0.29	0.53	-0.12	12%	3
Treg/Cytotoxic-Tumor Mixing	All	-0.09	0.15	0.02	-0.09	0.07	-0.44	0.82	0.06	-0.31	6%	4
	NSCLC	-0.04	0.12	0.08	-0.08	0.06	-0.44	0.81	0.08	-0.33	6%	4
	BRCA	-0.12	0.12	-0.13	0.07	-0.09	-0.42	0.69	0.30	-0.45	4%	6
	CRC	0.09	0.25	-0.16	0.16	-0.23	-0.45	0.70	0.21	-0.30	7%	4
	Other Cancer	-0.16	0.31	-0.01	-0.10	0.02	-0.43	0.80	0.05	-0.22	7%	4
	Low Stage	-0.01	-0.04	0.03	-0.06	0.11	-0.40	0.83	0.03	-0.36	7%	4
	High Stage	-0.13	0.27	0.00	-0.11	0.03	-0.44	0.79	0.09	-0.26	6%	4
	Genomics	0.00	-0.01	0.05	-0.05	0.10	-0.40	0.82	0.04	-0.39	6%	4
Treg/Cytotoxic Enrichment	All	-0.43	0.78	-0.09	-0.17	-0.08	-0.12	-0.13	0.05	0.37	5%	5
	NSCLC	-0.48	0.81	-0.18	-0.14	0.00	-0.05	-0.07	-0.01	0.24	5%	5
	BRCA	-0.50	0.79	-0.27	-0.06	0.06	0.13	-0.13	-0.06	0.04	3%	7
	CRC	-0.59	0.65	0.10	-0.24	0.08	0.30	0.04	0.06	-0.21	5%	6
	Other Cancer	-0.38	0.75	-0.04	-0.23	-0.08	-0.01	-0.29	0.02	0.38	5%	5
	Low Stage	-0.51	0.71	0.02	-0.17	-0.03	-0.25	0.06	-0.02	0.38	5%	5
	High Stage	-0.39	0.79	-0.14	-0.17	-0.08	0.03	-0.25	0.10	0.30	5%	5
	Genomics	-0.38	0.83	-0.16	-0.16	-0.11	-0.20	0.01	0.18	0.19	5%	5

Figure S2. Results of PCA performed on different case subsets. Related to Figure 2. PC rank indicates the original order of the PC in terms of explained variance for the subset. High consistency is observed across the case subsets in terms of both the PC weights and variance explained. 'Other Cancer' includes cases from any cancer other than NSCLC, BRCA, and CRC. Genomics includes cases for which genomic profiling is available. NSCLC: non-small cell lung cancer, BRCA: breast cancer, CRC: colorectal cancer, PC: principal component, PCA: principal component analysis.

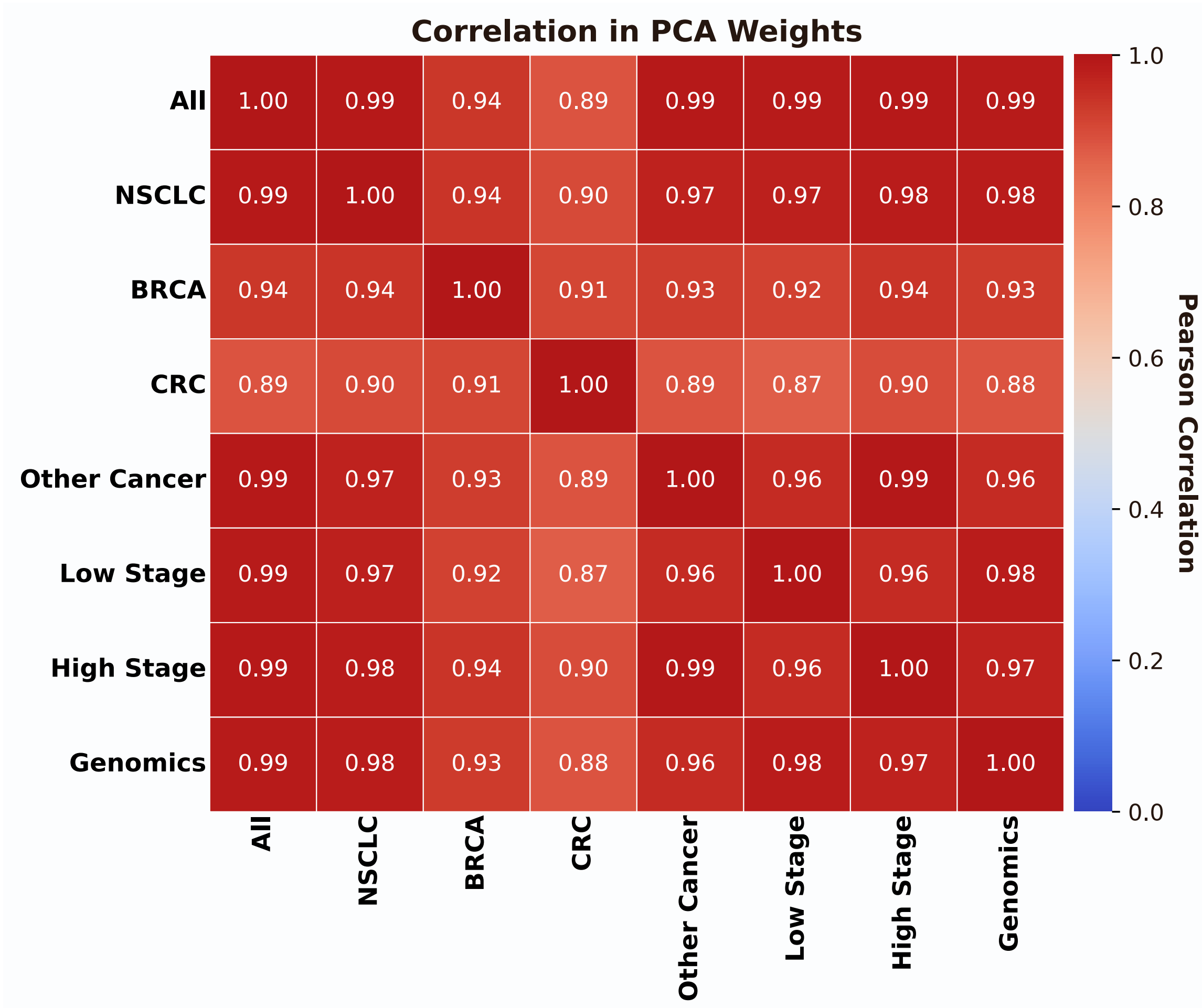

Figure S3. Correlation in principal component weights between different case subsets. Related to Figure 2. Correlation is computed between the five PCs representing the TIME spatial factors described in the text. High correlation is observed between each pair. ‘Other Cancer’ includes cases from any cancer other than NSCLC, BRCA, and CRC. Genomics includes cases for which genomic profiling is available. NSCLC: non-small cell lung cancer, BRCA: breast cancer, CRC: colorectal cancer, PC: principal component, PCA: principal component analysis.

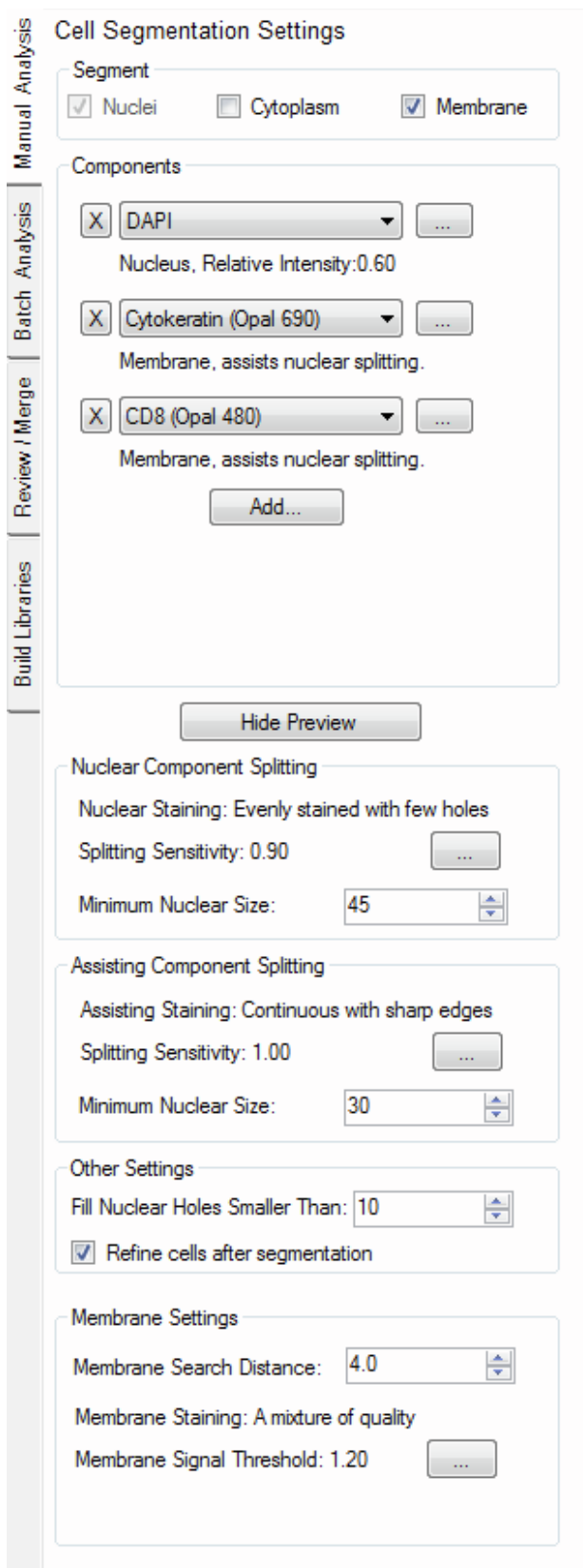

Figure S4. Default cell segmentation settings used in inForm. Related to STAR Methods. Cell segmentation was performed using a scientist-in-the-loop approach to refine the settings as necessary for a given case, followed by review and approval by a pathologist.

		Stage			Tissue Sample Type			Immunotherapy			Genomics	
**Cancer Type**	**Count (%)**	**Low**	**High**	**Unknown**	**Primary**	**Metastatic**	**Unknown**	**No**	**Yes**	**Unknown**	**No**	**Yes**
**Non-Small Cell Lung Cancer**	507 (25.1)	301 (59.4)	203 (40.0)	3 (0.6)	413 (81.5)	89 (17.6)	5 (1.0)	357 (70.4)	147 (29.0)	3 (0.6)	104 (20.5)	403 (79.5)
**Breast Cancer**	257 (12.7)	147 (57.2)	110 (42.8)	0 (0.0)	196 (76.3)	57 (22.2)	4 (1.6)	217 (84.4)	0 (0.0)	40 (15.6)	164 (63.8)	93 (36.2)
**Colorectal Cancer**	233 (11.5)	49 (21.0)	184 (79.0)	0 (0.0)	131 (56.2)	101 (43.3)	1 (0.4)	185 (79.4)	30 (12.9)	18 (7.7)	48 (20.6)	185 (79.4)
**Bladder Cancer**	160 (7.9)	101 (63.1)	59 (36.9)	0 (0.0)	139 (86.9)	21 (13.1)	0 (0.0)	107 (66.9)	53 (33.1)	0 (0.0)	89 (55.6)	71 (44.4)
**Esophagogastric Cancer**	123 (6.1)	35 (28.5)	84 (68.3)	4 (3.3)	91 (74.0)	31 (25.2)	1 (0.8)	83 (67.5)	20 (16.3)	20 (16.3)	46 (37.4)	77 (62.6)
**Melanoma**	108 (5.3)	23 (21.3)	84 (77.8)	1 (0.9)	42 (38.9)	65 (60.2)	1 (0.9)	10 (9.3)	98 (90.7)	0 (0.0)	31 (28.7)	77 (71.3)
**Pancreatic Cancer**	104 (5.2)	31 (29.8)	73 (70.2)	0 (0.0)	49 (47.1)	55 (52.9)	0 (0.0)	89 (85.6)	9 (8.7)	6 (5.8)	23 (22.1)	81 (77.9)
**Head and Neck Cancer**	95 (4.7)	38 (40.0)	55 (57.9)	2 (2.1)	76 (80.0)	19 (20.0)	0 (0.0)	53 (55.8)	40 (42.1)	2 (2.1)	61 (64.2)	34 (35.8)
**Endometrial Cancer**	94 (4.7)	55 (58.5)	38 (40.4)	1 (1.1)	77 (81.9)	17 (18.1)	0 (0.0)	75 (79.8)	4 (4.3)	15 (16.0)	49 (52.1)	45 (47.9)
**Ovarian Cancer**	92 (4.6)	18 (19.6)	73 (79.3)	1 (1.1)	42 (45.7)	50 (54.3)	0 (0.0)	78 (84.8)	3 (3.3)	11 (12.0)	30 (32.6)	62 (67.4)
**Renal Cell Carcinoma**	83 (4.1)	23 (27.7)	60 (72.3)	0 (0.0)	43 (51.8)	40 (48.2)	0 (0.0)	44 (53.0)	39 (47.0)	0 (0.0)	51 (61.4)	32 (38.6)
**Prostate Cancer**	35 (1.7)	8 (22.9)	27 (77.1)	0 (0.0)	19 (54.3)	16 (45.7)	0 (0.0)	27 (77.1)	8 (22.9)	0 (0.0)	12 (34.3)	23 (65.7)
**Hepatobiliary Cancer**	33 (1.6)	12 (36.4)	20 (60.6)	1 (3.0)	26 (78.8)	7 (21.2)	0 (0.0)	23 (69.7)	4 (12.1)	6 (18.2)	10 (30.3)	23 (69.7)
**Small Cell Lung Cancer**	20 (1.0)	5 (25.0)	15 (75.0)	0 (0.0)	12 (60.0)	7 (35.0)	1 (5.0)	13 (65.0)	7 (35.0)	0 (0.0)	10 (50.0)	10 (50.0)
**Other**	75 (3.7)	4 (5.3)	35 (46.7)	36 (48.0)	37 (49.3)	30 (40.0)	8 (10.7)	39 (52.0)	4 (5.3)	32 (42.7)	16 (21.3)	59 (78.7)
**Total**	2019 (100.0)	850 (42.1)	1120 (55.5)	49 (2.4)	1393 (69.0)	605 (30.0)	21 (1.0)	1400 (69.3)	466 (23.1)	153 (7.6)	744 (36.8)	1275 (63.2)

Table S1. Distribution of cases in cohort. Related to Figure 1. Tissue sample type indicates whether the specimen represents a primary or metastatic site. Genomics indicates the availability of genomic profiling. Immunotherapy indicates whether the patient was treated with immunotherapy either before or after the specimen date. Cancer types with less than 20 cases were grouped as 'Other' and include the following: Cancer of Unknown Primary (17 cases), Cervical Cancer (12), Thyroid Cancer (11), Anal Cancer (8), Gastrointestinal Neuroendocrine Tumor (5), Small Bowel Cancer (5), Salivary Gland Cancer (5), Non-Melanoma Skin Cancer (4), Mesothelioma (3), Ampullary Cancer (3), Vaginal Cancer (1), Thymic Carcinoma (1).

	Analysis Reproducibility (R^2)	
**Biomarker Metric**	**Same ROIs**	**Independent ROIs**
CD8 Density	0.96	0.90
FOXP3 Density	0.99	0.75
PD-1 Density	0.75	0.76
PD-L1 TPS	0.99	0.94
PD-L1 IC	0.86	0.80

Table S2. Image Analysis Reproducibility Results. Related to STAR Methods. Two scientists independently performed the image analysis for a set of 20 cases, once using the same ROIs and once with independently selected ROIs. TPS: tumor proportion score; IC: immune score (% of intratumor area occupied by PD-L1+ immune cells).
