# Supplementary material for: Pan-cancer spatial characterization of key immune biomarkers in the tumor microenvironment
Source: Cell Rep Med. 2025 Oct 14;7(10):102418. doi: 10.1016/j.xcrm.2025.102418 (PMC13710152; doi:10.1016/j.xcrm.2025.102418)
Supplement: Supplementary file 2 [file mmc5.pdf]

Cell Reports
Medicine

Article

Pan-cancer spatial characterization of key immune biomarkers in the tumor microenvironment

Graphical abstract

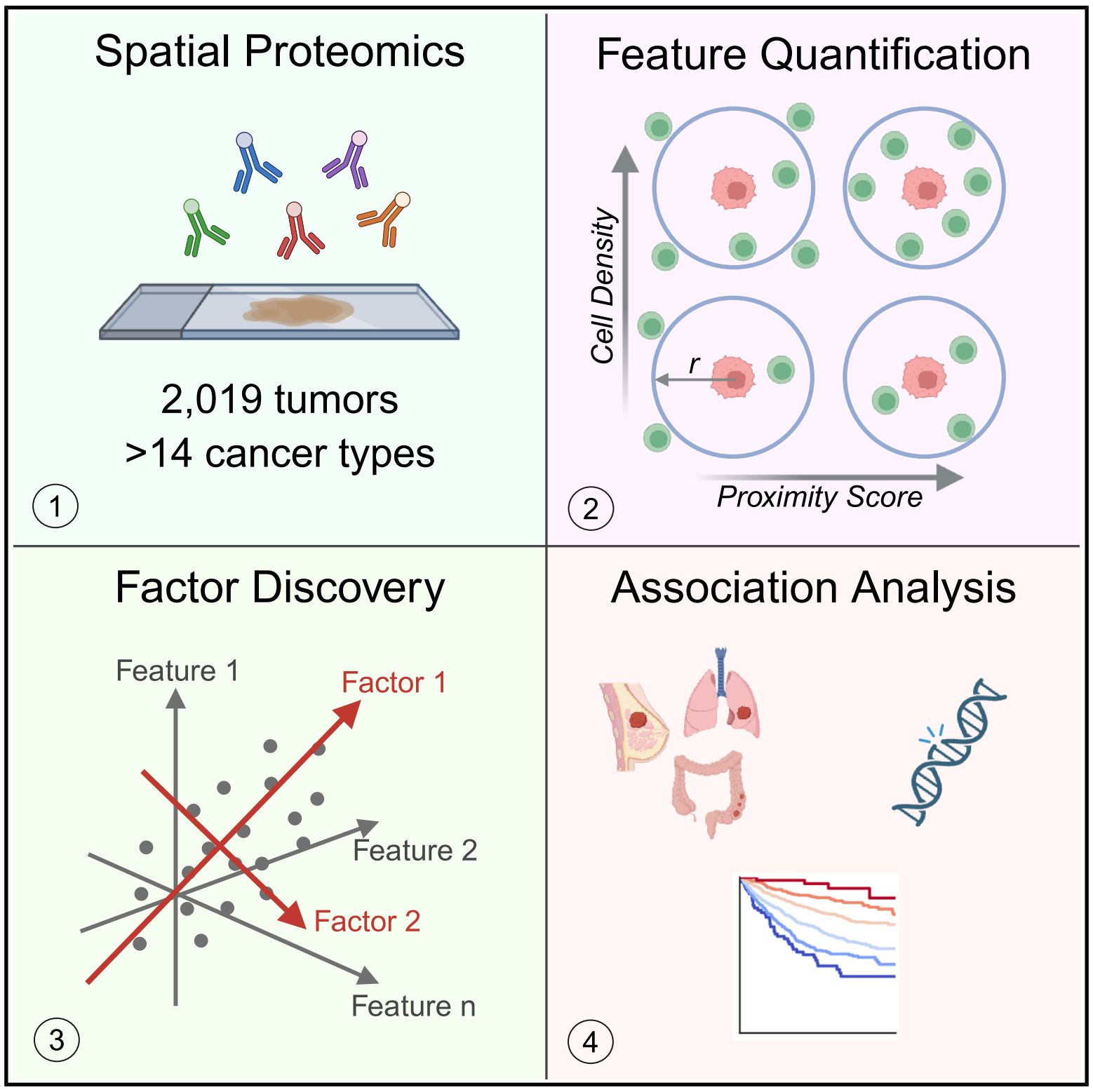

Authors

James R. Lindsay, Jennifer Altreuter, Joao V. Alessi, ..., Mark M. Awad, Scott J. Rodig, William Lotter

Correspondence

lotterb@ds.dfci.harvard.edu

In brief

Lindsay et al. present a pan-cancer spatial characterization of the tumor immune microenvironment using multiplex immunofluorescence data from 2,019 tumors. Spatial factors conserved across cancer types are identified that correlate with genomics and patient outcomes. A spatially resolved single-cell database comprising 39.4 million cells accompanies the findings.

Highlights

- Spatial proteomics analysis of a prospective pan-cancer cohort of 2,019 tumors
- Identification of spatial factors conserved across cancer types and stages
- Spatial factors are linked to genomic alterations and patient outcomes
- Generation of a spatially resolved single-cell database comprising 39.4 million cells

Lindsay et al., 2026, Cell Reports Medicine 7, 102418
October 20, 2026 © 2025 The Author(s). Published by Elsevier Inc.
https://doi.org/10.1016/j.xcrm.2025.102418

CellPress

Cell Reports Medicine

CellPress OPEN ACCESS

Article

Pan-cancer spatial characterization of key immune biomarkers in the tumor microenvironment

James R. Lindsay,[1] Jennifer Altreuter,[1] Joao V. Alessi,[2,6] Jason L. Weirather,[1,3] Anita Giobbie-Hurder,[1,3] Ian Dryg,[1,3] Katharina Hoebel,[1,4] Bijaya Sharma,[3] Kristen Felt,[3] F. Stephen Hodi,[2,3] Neal Lindeman,[5,7] Lynette M. Sholl,[5] Ethan Cerami,[1] Jonathan A. Nowak,[4,5] Mark M. Awad,[2,8] Scott J. Rodig,[3,5,9] and William Lotter[1,4,5,9,10,*]

[1]Department of Data Science, Dana-Farber Cancer Institute, Boston, MA, USA
[2]Department of Medical Oncology, Dana-Farber Cancer Institute, Boston, MA, USA
[3]Center for Immuno-Oncology, Dana-Farber Cancer Institute, Boston, MA, USA
[4]Harvard Medical School, Boston, MA, USA
[5]Department of Pathology, Brigham & Women's Hospital, Boston, MA, USA
[6]Present address: Hospital Sirio Libanes, São Paulo, Brazil
[7]Present address: Weill Cornell Medical College, New York, NY, USA
[8]Present address: Memorial Sloan Kettering Cancer Center, New York, NY, USA
[9]Senior author
[10]Lead contact
*Correspondence: lotterb@ds.dfci.harvard.edu
https://doi.org/10.1016/j.xcrm.2025.102418

SUMMARY

Deciphering the composition and spatial organization of the tumor immune microenvironment (TIME) is key to understanding cancer progression and treatment response. Spatial biology techniques such as multiplex immunofluorescence (mIF) offer detailed insights but are often limited to retrospective studies of individual cancer types. Here, we provide a pan-cancer spatial characterization of key biomarkers (CD8, FOXP3, PD-1, and PD-L1) using an mIF assay performed prospectively in a clinical setting on 2,019 tumors across 14 major cancer types. By integrating interpretable compositional and spatial metrics, we identify patterns of TIME variation conserved across cancer types and stages. We assess associations between these TIME spatial factors and tumor, genomic, and clinical features, with results extending prior findings and uncovering new links. To accompany the analysis, we provide a curated database of the 39.4 million spatially resolved cells. Altogether, our findings and database offer pan-cancer insights of the TIME to advance spatial biology and cancer immunology.

INTRODUCTION

Tumors develop in complex microenvironments containing a variety of immune and stromal cells that can both enhance and suppress cancer progression. Understanding the cellular composition and spatial organization of these tumor immune microenvironments (TIMEs) is important for developing more effective therapeutic strategies.[1–7] To this end, spatial biology techniques, such as multiplex immunofluorescence (mIF), provide detailed views of the TIME by measuring biomarkers in tissue samples while preserving spatial context.[1,2,8–15] Studies using mIF imaging have demonstrated associations between TIME features and patient outcomes,[8–10,16–18] such as the relationship between intratumoral CD8$^+$ and FOXP3$^+$ cell densities and prognosis in lung[10,17] and colorectal cancers.[18] However, most mIF studies to date have focused on individual cancer types in pre-selected populations. Furthermore, important opportunities remain to explore how mIF-derived spatial features correlate with the genomic phenotypes of tumors.

Here, we characterize spatial aspects of the TIME based on a standardized mIF assay[19] that was performed prospectively in a clinical setting on tumor tissue specimens from 2,019 tumors across over 14 cancer types (Figure 1). The assay profiles four key immune biomarkers (CD8, FOXP3, PD-1, and PD-L1), a tumor cell marker, and DAPI for nuclei detection. By combining metrics of cellular composition and spatial organization, we first aimed to identify the major patterns of TIME variation across the cohort. We then quantified how these patterns associate with tumor, genomic, and clinical features. To facilitate further efforts, we have also released a spatially resolved, single cell database of the 39.4 million cells in the cohort with accompanying clinical and genomic annotations.

RESULTS

Pan-cancer spatial atlas of the tumor immune microenvironment

The mIF assay, named ImmunoProfile, was performed prospectively in a CLIA-certified laboratory on tumor tissue specimens

© 2025 The Author(s). Published by Elsevier Inc.
This is an open access article under the CC BY license (http://creativecommons.org/licenses/by/4.0/).

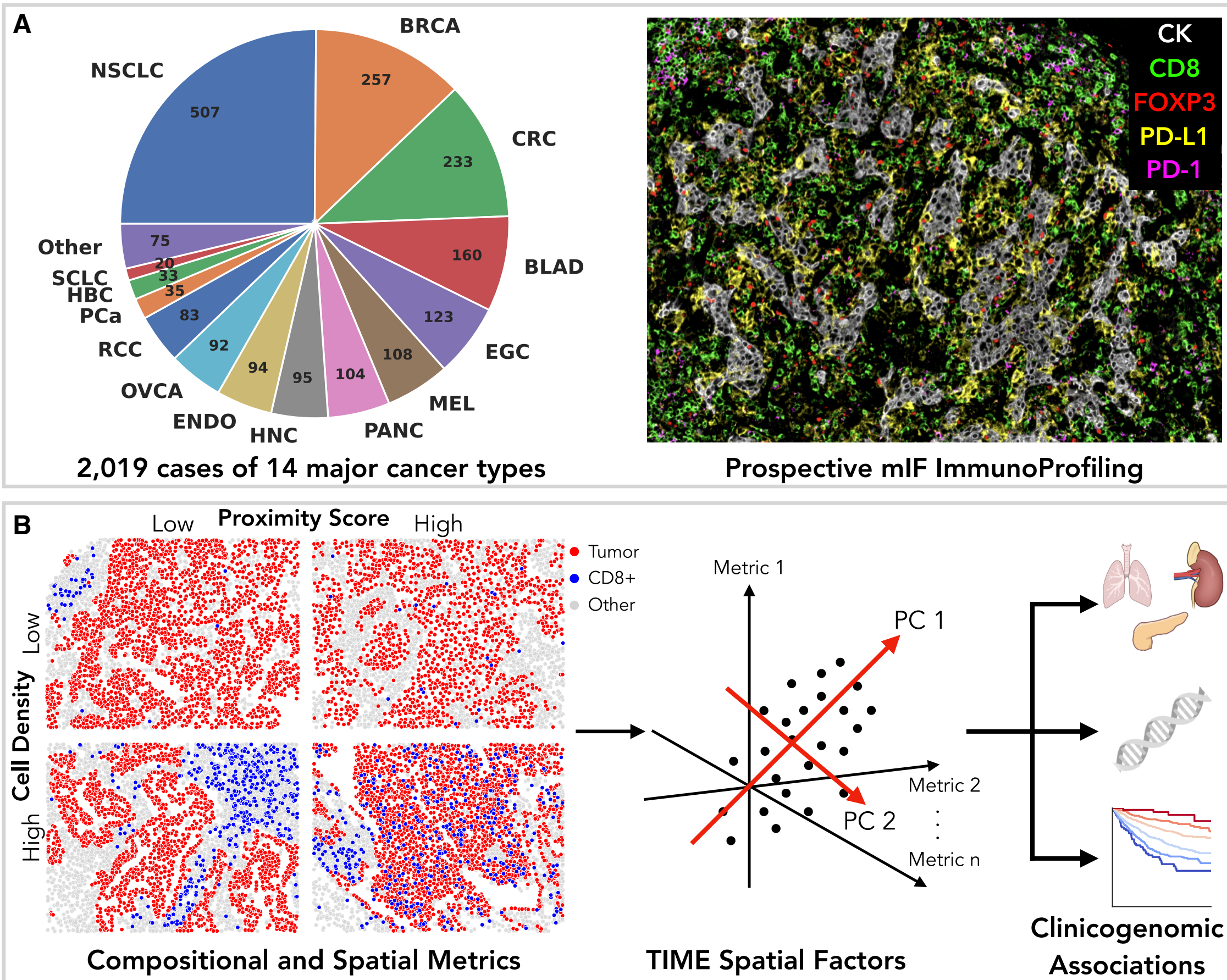

Figure 1. Pan-cancer spatial characterization of the TIME

(A) An mIF assay (ImmunoProfile) was performed prospectively across 2,109 cases representing 14 major cancer types. The distribution of cancer types and an example ROI are displayed.

(B) Summary of computational analysis. Cellular compositional and spatial metrics were computed, including the density of cells positive for each immune biomarker and a "proximity score" that quantifies the relative proximity of biomarker positive cells to tumor cells. The metrics are illustrated for $CD8^+$ cells using four NSCLC ROIs, where dots indicate the location of individual cells. The metrics across biomarkers were combined to identify distinct "TIME spatial factors" through principal-component analysis. Associations were then assessed between the TIME spatial factors and tumor, genomic, and clinical variables.

NSCLC, non-small cell lung cancer; BRCA, breast cancer; CRC, colorectal cancer; BLAD, bladder cancer; EGC, esophagogastric cancer; MEL, melanoma; PANC, pancreatic cancer; HNC, head and neck cancer; ENDO, endometrial cancer; OVCA, ovarian cancer; RCC, renal cell carcinoma; PCa, prostate cancer; HBC, hepatobiliary cancer; SCLC, small cell lung cancer; PC, principal component. See also Table S1.

from consenting patients over a three-year period at the Dana-Farber Cancer Institute (DFCI).[19,20] The cohort includes 14 major cancer types as organized by OncoTree[21] diagnosis codes (Figure 1A). Among these, 10 cancer types have over 90 cases each. At the time of specimen collection, 850 (42%) cases were low stage (stage II or below), 1,120 (55%) cases were high stage (stage III or above), and 49 (2.4%) were of unknown stage. For each case, an mIF whole slide image (WSI) was generated at 20× resolution with staining performed on a section from a formalin-fixed, paraffin-embedded (FFPE) tissue specimen. Intratumoral regions of interest (ROIs) (Figure 1B) were selected by trained scientists and pathologists for each mIF WSI based on a standardized protocol. For each ROI, the *x*-*y* coordinates of cells positive for each biomarker were extracted using inForm software (Akoya Biosciences, Marlborough, MA, USA) in conjunction with custom scripts and approval by a pathologist. Several validation and reproducibility studies were performed that support the robustness of the ImmunoProfile assay and analysis pipeline.[20] This includes a validation study comparing the mIF-derived cell densities to those quantified based on immunohistochemistry (IHC), the gold standard for biomarker staining and quantification. Matched ROIs from IHC and mIF images from serial sections of 10 cases (9 cancer cases, 1 normal tonsil tissue) were analyzed, demonstrating high concordance between IHC and mIF for each biomarker with R^2 values > 0.99 (CD8: 0.9955, PD-1: 0.9961, FOXP3: 0.9960, PD-L1: 0.9978, and CK: 0.9984). Further details on the assay and its validation are contained in the STAR Methods.

Genetic profiling was available for 1,275 (63%) cases via a targeted next-generation sequencing assay performed as part of the Profile program.[22,23] In total, the analyzed cohort contains 39.4 million detected cells across 2,019 unique patients

CellPress
OPEN ACCESS

(Table S1). Representing an unselected patient population with standard-of-care clinical histories, the cohort provides a unique opportunity to describe key aspects of the TIME in a real-world clinical setting.

Identifying TIME spatial factors

We first sought to describe the major patterns of TIME variation in the cohort by generating quantitative metrics of the cellular composition and spatial organization for each case. For cell composition, we use the density of cells positive for each immune biomarker (CD8, FOXP3, PD-1, and PD-L1) within the annotated intratumoral ROIs, and the PD-L1 tumor proportion score (TPS) as a tumor-specific metric. When computing cell densities for the immune biomarkers, cells positive for the biomarker and negative for the tumor marker were included. To quantify spatial organization, we derived a spatial metric that measures the relative proximity of a given cell type to tumor cells in an ROI while controlling for the density of the cell type (Figure 1B). This metric leverages the spatially resolved data to quantify the likelihood that two cells are in close proximity within an ROI relative to a random distribution, calculated using a formulation based on the G-cross function[24,25] (see STAR Methods). We refer to this metric as the "proximity score." In contrast to common spatial metrics[26] such as nearest neighbor distance, the proximity score is designed to have low correlation with cell density to enable insights into differences between spatial structure versus overall composition. We compute the proximity score separately for cells positive for each immune biomarker (CD8, FOXP3, PD-1, and PD-L1) in reference to tumor cells.

With the goal of identifying distinct patterns of variation across the pan-cancer cohort, we first assessed the pairwise correlations between all of the cell density and proximity score metrics. We observe low correlations between the proximity scores and cell densities as intended (Figure S1), but moderate correlations exist between the different biomarkers within each metric. For instance, tumors with high $CD8^+$ cell densities also tended to have higher $FOXP3^+$ cell densities (Spearman correlation of 0.60 [95% confidence interval (CI): 0.56, 0.63]). Similarly, tumors with higher CD8 proximity scores tended to have higher FOXP3 proximity scores (Spearman correlation of 0.47 [95% CI: 0.44, 0.51]). To account for these correlations, we performed principal-component analysis (PCA). PCA identifies new uncorrelated variables (principal components [PCs]) that are linear combinations of the original variables, such that the first PC captures the maximum variance across the dataset, the second captures the next highest variance orthogonal to the first, and so on. Each PC is defined by a set of weights across the original variables and explains a specific percentage of the total variance in the dataset.

The results of the PCA decomposition for the 2,019-patient ImmunoProfile cohort are illustrated in Figure 2. The number of PCs was chosen to approximate 90% variance explained across the cohort, resulting in five PCs (89% total variance explained). Figure 2A displays the variance explained and weights for these PCs, where weights above one standard deviation (SD) are highlighted in green and weights below negative one SD are highlighted in red. The first PC contains high weights for each of the immune biomarker cell densities and TPS. Tumors that score high for this "immune enrichment" factor demonstrate an inflamed ("hot") phenotype, with low scoring cases indicating a "cold" phenotype. The second PC has high weights for the proximity scores across the immune biomarkers. Tumors scoring high for this "immune-tumor mixing" factor demonstrate a spatially "mixed" phenotype, whereas low scoring cases demonstrate an "excluded" phenotype. The third PC indicates an "immune evasion" signature. Tumors scoring high for this factor have high TPS, high $PD\text{-}L1^+$ cell proximity to tumor cells, and low $CD8^+$ cell density. The fourth PC quantifies differences between the FOXP3 and CD8 proximity scores ("Treg/cytotoxic-tumor mixing") with higher values indicating relatively closer proximity of $FOXP3^+$ cells to tumor cells than $CD8^+$ cells to tumor cells. Analogously, PC5 largely quantifies differences between the FOXP3 and CD8 cell densities ("Treg/cytotoxic enrichment"). A moderately positive weight for the PD-1 proximity score is also present in PC5. Figure 2B contains example ROIs scoring low and high for each of the five PCs, which we term "TIME spatial factors."

Association of TIME spatial factors with cancer type and stage

As aspects of the TIME are generally thought to vary by cancer type, it is possible that different TIME spatial factors would be identified if the analysis was performed for specific cancer types rather than for the entire pan-cancer cohort. Alternatively, the TIME spatial factors themselves may be conserved, but the distribution of the factors may differ by cancer type (e.g., a higher proportion of "hotter" tumors in some cancers). These considerations also extend to cancer stage, where high-stage tumors may reflect a failure of the immune system to control tumor growth.

To assess the possibility that different TIME spatial factors would be identified for different case subsets, we performed PCA separately for cancer types with over 200 cases (non-small cell lung cancer [NSCLC], breast cancer, and colorectal cancer [CRC]), cases from the remaining cancer types, low stage cases, high stage cases, and cases with genomic profiling available. We observe similar PCA weights and variance percentages for each of these subsets (Figures S3 and S2). The correlation (*r*) in PCA weights between each subset and the entire cohort ranged from 0.89 to 0.99 (Figure S3). These results suggest that the five TIME spatial factors described previously are generally representative of the patterns of variation across the pan-cancer cohort.

To assess whether the distribution of the TIME spatial factors varies across case subsets, we first computed scores for the TIME spatial factors for each case by applying the PCA weights. We then fit a linear mixed model[27] to estimate the percentage of the variance in these scores that is attributable to cancer type and stage (Figure 3B). The attributable variance ranged from 4.2% (Treg/cytotoxic-tumor mixing [PC4]) to 18.8% (immune enrichment [PC1]), indicating that that the majority (81.2%–95.8%) of the variability in the TIME spatial factor scores across the cohort is not explained by cancer type or stage. The variance specifically attributable to cancer type ranged from 3.4% (immune evasion [PC3]) to 17.8% (Treg/cytotoxic enrichment

A

	Cell Density				Tumor	Proximity Score				% Variance Explained
PC #	CD8	FOXP3	PD-L1	PD-1	TPS	CD8	FOXP3	PD-L1	PD-1	
1	0.41	0.36	0.44	0.42	0.39	0.22	0.16	0.27	0.21	43%
2	-0.18	-0.28	-0.20	-0.22	-0.05	0.49	0.44	0.33	0.50	24%
3	-0.33	-0.21	0.21	-0.28	0.54	-0.20	-0.28	0.54	-0.13	11%
4	-0.09	0.15	0.02	-0.09	0.07	-0.44	0.82	0.06	-0.31	6%
5	-0.43	0.78	-0.09	-0.17	-0.08	-0.12	-0.13	0.05	0.37	5%

B

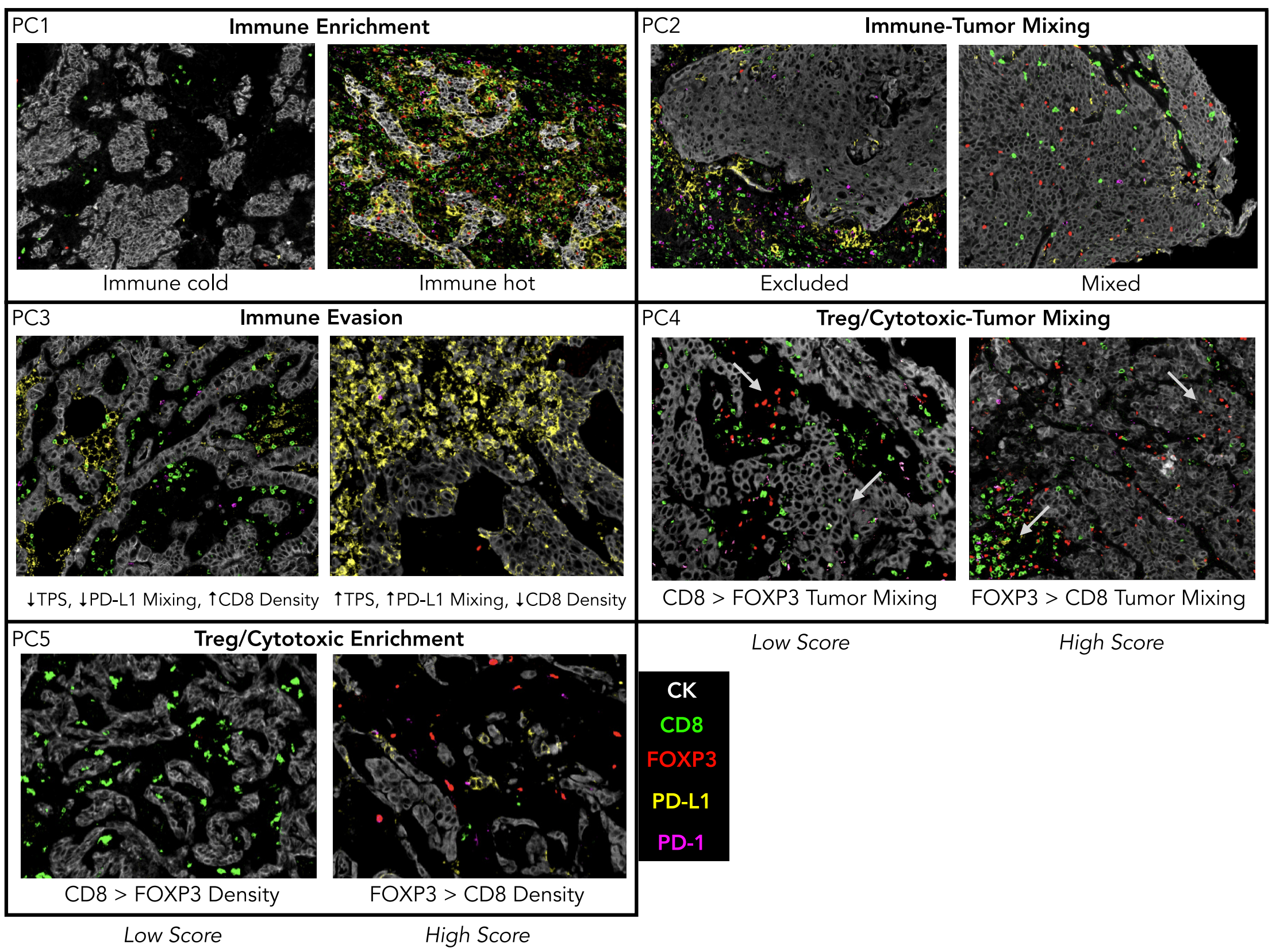

Figure 2. Spatial factors of the tumor immune microenvironment

(A) PCA weights and corresponding attributable variance across the cohort.

(B) Illustration of the TIME spatial factors. mIF image patches from NSCLC ROIs scoring high and low for each factor are displayed.

PC, principal component; TPS, PD-L1 tumor proportion score. See also Figures S1, S2, and S3.

[PC5]), and from 0.2% (Treg/cytotoxic-tumor mixing [PC4]) to 5.5% (immune enrichment [PC1]) for stage.

To visualize trends in the TIME spatial factor scores for specific cancer types/stages, we binarized the scores into high vs. low based on a median split across the cohort, followed by computing the percentage of high scores for each subset (Figure 3A). For the immune enrichment (PC1) factor, the percentage of high scoring cases per cancer type ranged from 24.9% (breast cancer) to 81.9% (head and neck cancer). Other hotter cancer types included NSCLC, melanoma, and renal cell carcinoma (RCC); other colder cancer types included prostate, pancreatic, and bladder. For Treg/cytotoxic enrichment (PC5), which varied the most by cancer type, head and neck cancer had the highest proportion of high values (i.e., more FOXP3$^+$ cell enriched), followed by CRC and esophagogastric cancer; the lowest proportions (i.e., more CD8$^+$ cell enriched) were observed for RCC, endometrial cancer, and small cell lung cancer. Figure 3C visualizes the immune enrichment and Treg/cytotoxic enrichment factors in combination per cancer type, where each combination of hotter/colder and more FOXP3 enriched/more CD8 enriched is represented. For cancer stage, high stage tumors had a slight trend toward higher scores for immune evasion (PC3) and lower scores for immune enrichment (PC1) (Figure 3A). Altogether, while a fraction (<20%) of the variance in the TIME spatial factors is attributable to cancer type and stage, high and low values for each factor are present for each subset, further supporting the generality of the factors.

Association of TIME spatial factors with genomic alterations

Along with cancer type and stage, the genomic phenotype of a tumor may correlate with different aspects of the TIME. We

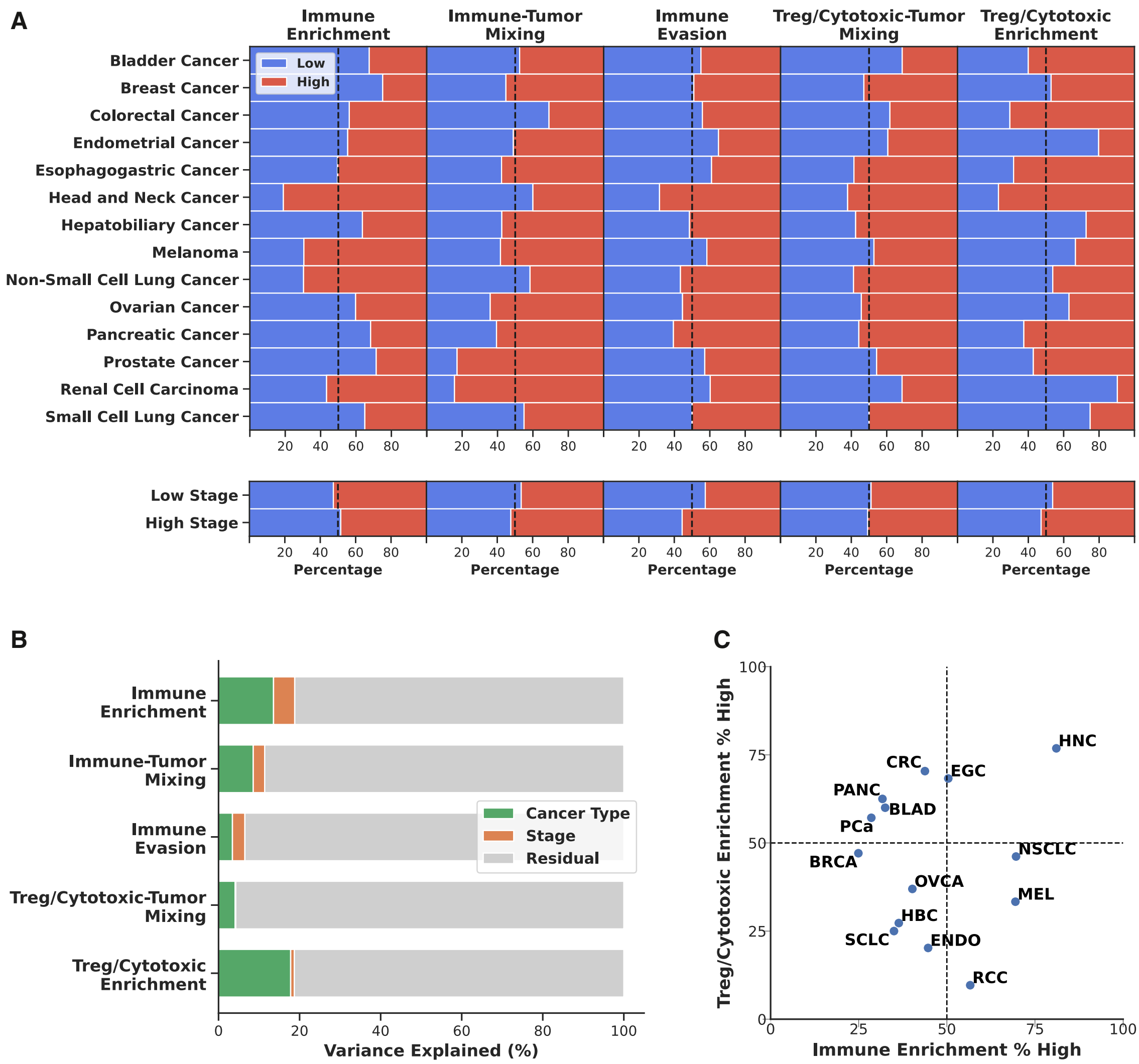

Figure 3. Distribution of TIME spatial factors by cancer type and stage

(A) Percentage of high and low scoring cases for each factor by cancer type and stage based on a median split across the cohort.

(B) Variance attributable to cancer type and stage for each factor.

(C) Distribution of cancer types according to the immune enrichment and Treg/cytotoxic enrichment factors, which varied the most by cancer type. Axes represent the percentage of cases scoring high for the respective factor.

NSCLC, non-small cell lung cancer; BRCA, breast cancer; CRC, colorectal cancer; BLAD, bladder cancer; EGC, esophagogastric cancer; MEL, melanoma; PANC, pancreatic cancer; HNC, head and neck cancer; ENDO, endometrial cancer; OVCA, ovarian cancer; RCC, renal cell carcinoma; PCa, prostate cancer; HBC, hepatobiliary cancer; SCLC, small cell lung cancer.

assessed associations between the TIME spatial factors and genomic alterations at three levels: somatic mutations, copy number alterations (CNAs), and tumor mutational burden (TMB). This analysis was performed for the 1,275 (63%) tumors in the cohort with available genomic data. Associations were adjusted for cancer type and multiple hypothesis testing (Benjamini-Hochberg correction[28] with a false discovery rate of 10%). For somatic mutations and CNAs, only alterations that were present in at least five cancer types and that were annotated as “oncogenic” or “likely oncogenic” by OncoKB[29,30] were included.

Figure 4 summarizes the associations between the TIME spatial factors and somatic mutations. Immune enrichment (PC1) shows the largest number of associations, including positive associations with mutations in DNA damage repair genes such as *ATR*, *BRCA1*, *MLH3*, *MSH6*, *PMS2*, and *RAD50*. The strongest negative association with immune enrichment was observed for *EGFR* (T = −3.61, p = 0.008). The remaining factors each show an association with at least one gene: immune-tumor mixing (PC2) is negatively associated with *APC* (T = −3.95, p = 0.008), immune evasion (PC3) is positively associated with *TP53* (T = 4.32, p = 0.002) and negatively with *CDH1* (T = −3.22, p = 0.066), Treg/cytotoxic-tumor mixing (PC4) is negatively associated with *ATRX* (T = −3.13, p = 0.095), and Treg/cytotoxic enrichment (PC5) is positively associated with *TP53* (T = 3.37, p = 0.077).

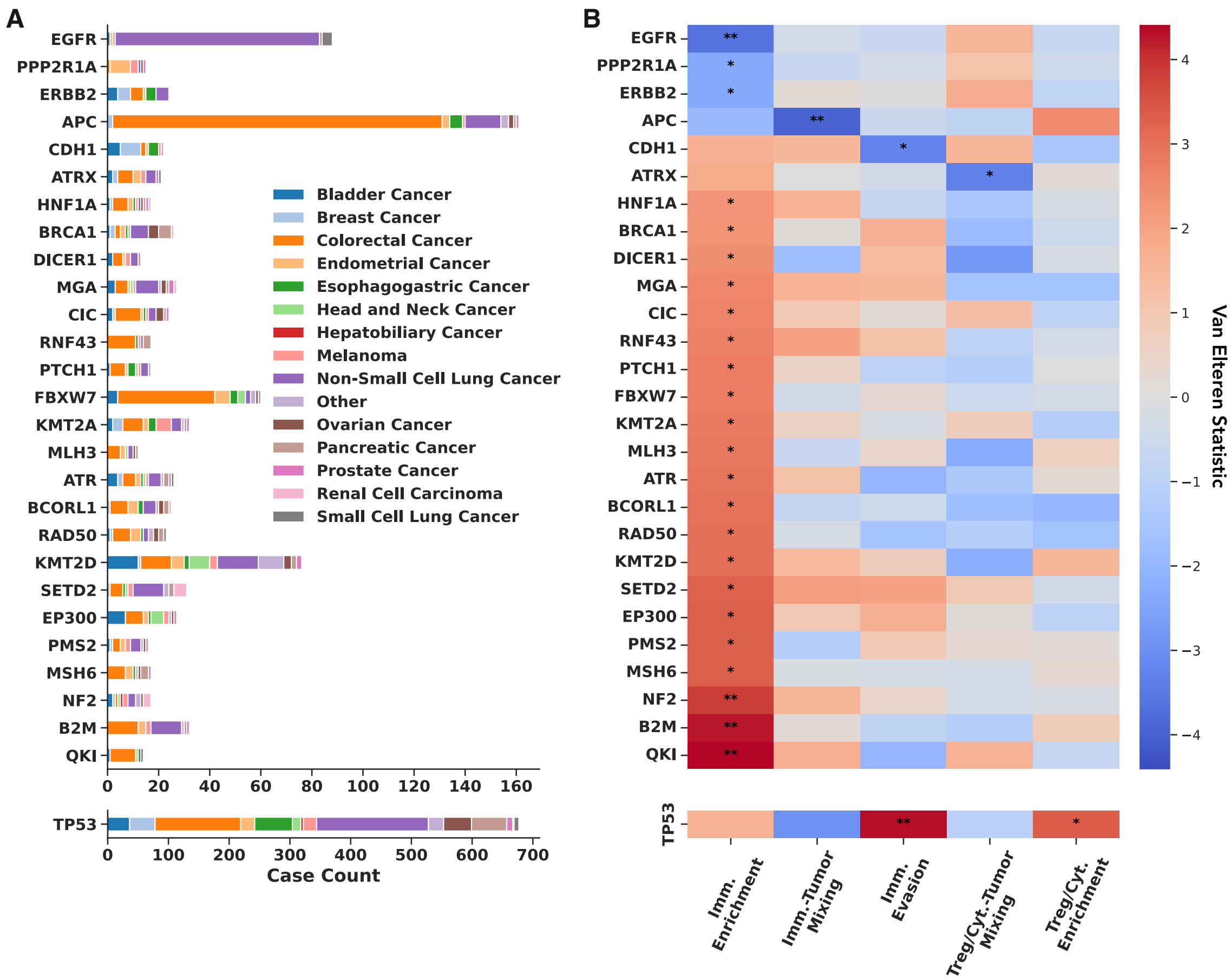

Figure 4. Associations between TIME spatial factors and somatic mutations

(A) Counts of mutated genes by cancer type. *TP53* is shown on a separate axis given high prevalence.

(B) Van Elteren T-statistic per gene and TIME spatial factor. Genes with a significant association with at least one factor are displayed based on a Benjamini-Hochberg correction with a false discovery rate of 10%.

$^{*}p < 0.1$ and $^{**}p < 0.01$.

Associations between the TIME spatial factors are also observed at the CNA level (Figure 5). Both immune enrichment (PC1) and immune evasion (PC3) are associated with multiple CNA events. Gains in arm 9p24.1 genes *CD274*, *JAK2*, and *PDCD1LG2* had some of the strongest positive associations with immune enrichment (T = 2.54, 2.30, 2.68, p = 0.044, 0.070, 0.032, respectively), while gains in 20q13.2 genes *ZNF217* and *AURKA* had the strongest negative associations (T = −6.21, −6.00, p = 3.8e−8, 7.3e−8) (Figure 5B). Only negative associations were observed between CNA loss events and immune enrichment, with loss of arm 9p21.3 genes *MTAP*, *CDKN2A*, and *CDKN2B* (T = −4.85, −4.74, −4.21, p = 1.3e−4, 1.8e−4, 0.001) having the strongest negative associations (Figure 5D). Strong immune evasion associations include gains in arm 3q genes *BCL6* and *PIK3CA* (T = 4.68, 4.39, p = 2.1e−4, 4.1e−4) and arm 9p24.1 genes *CD274*, *JAK2*, and *PDCD1LG2* (T = 3.70, 2.99, 3.70, p = 0.004, 0.040, 0.004). Immune-tumor mixing (PC2) is negatively associated with gains in 20q arm genes *ZNF217* and *AURKA* and 13q arm gene *CDK8* (T = −3.14, −2.92, −3.00, p = 0.08, 0.08, 0.08) (Figure 5D). Data S1, S2, and S3 contain results for all assessed mutations and CNAs.

Finally, for TMB, we find that the immune enrichment (PC1) factor has a positive association with high TMB status (≥10 mutations/Mb; T = 4.75, p = 1.0e−5) and the Treg/cytotoxic-tumor mixing (PC4) factor has a negative association (T = −2.79, p = 0.013). The remaining factors do not show significant associations with TMB (immune-tumor mixing (PC2): T = −2.53, p = 0.80; immune evasion (PC3): T = 1.33, p = 0.31; Treg/cytotoxic enrichment (PC5): T = −0.92, p = 0.45).

Association of TIME spatial factors with patient prognosis

We next explored associations between the TIME spatial factors and patient overall survival (OS). Figure 6A displays the concordance index (C-index) for each factor across the entire cohort (adjusted for cancer type and stage), per cancer type (adjusted for stage), and per stage (adjusted for cancer type). C-index values above 0.50 indicate positive correlation with survival, values below 0.50 indicate negative correlation with survival,

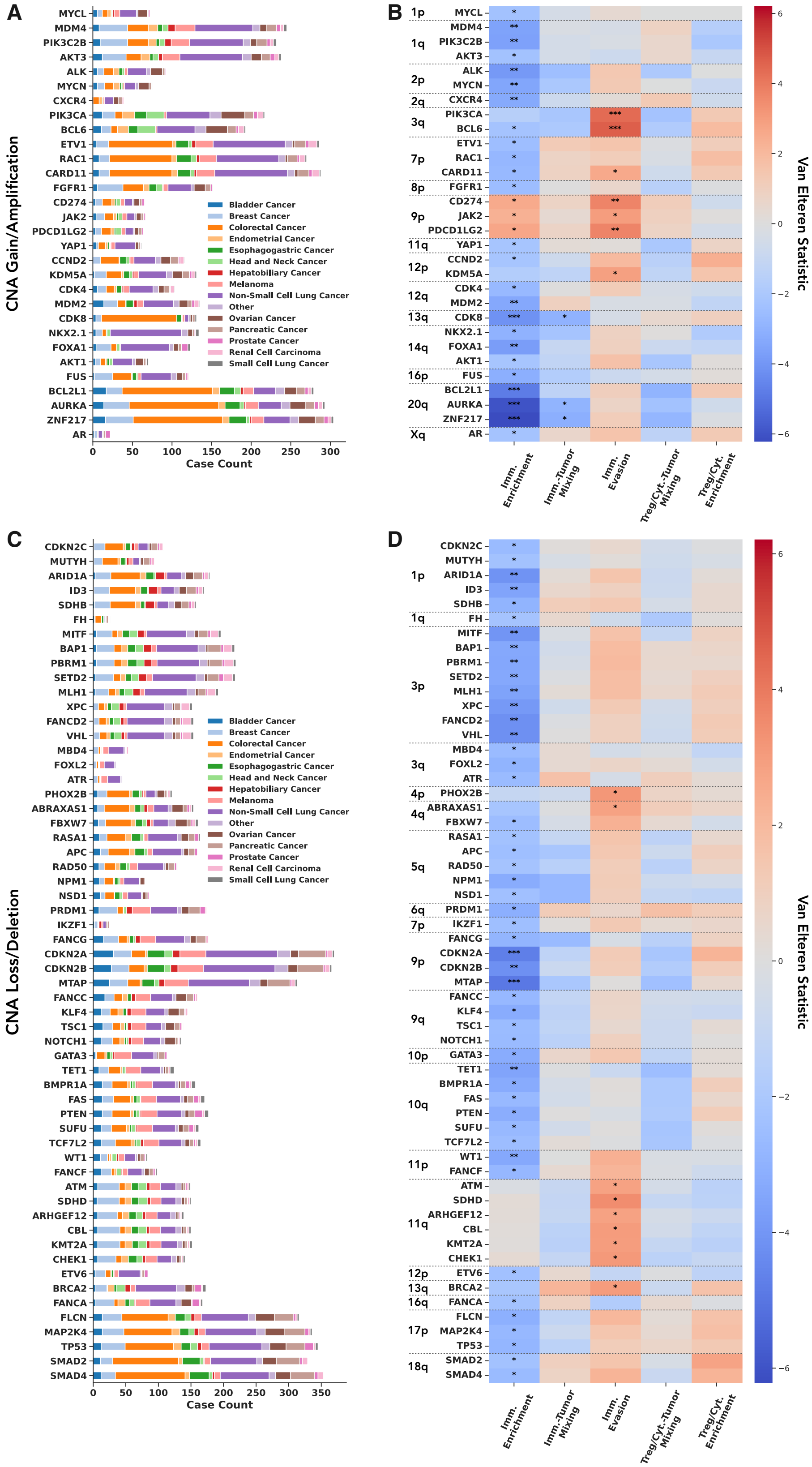

(legend on next page)

and a value of 0.50 indicates a chance result. The most prognostic factor across the cohort is immune evasion (PC3) with a C-index of 0.40 ($p = 3.1e-7$), signifying that higher scores for this factor are associated with reduced survival. This trend is consistent across cancer types and stages. For a sense of magnitude, tumors that score high for immune evasion have a 67% increased risk compared to those that score low (hazard ratio [HR] of 1.67 [95% CI: 1.41, 1.99] when binarized into "high" vs. "low" as in Figure 3A). The second most prognostic factor is immune enrichment (PC1) with a C-index of 0.57 ($p = 2.4e-6$; HR of 0.70 [95% CI: 0.59, 0.83] when binarized into high vs. low), indicating a positive association with survival. This association appears to be largely driven by high stage tumors, as an association is not observed for low stage tumors (Figure 6A). The remaining factors (immune-tumor mixing [PC2], Treg/cytotoxic-tumor mixing [PC4], Treg/cytotoxic enrichment [PC5]) all have negative associations with survival at the cohort level. For individual cancer types, there is some variation in the direction and magnitude of the C-index per factor, but all significant associations have consistent directions across the cohort.

To explore multi-factor associations with prognosis, we constructed a composite "TIME Score" that combines all of the TIME spatial factors. Each factor was first binarized as high vs. low based on median split across the cohort. For factors positively associated with survival (i.e., immune enrichment [PC1]), tumors with high scores were assigned a value of 1 and those with low scores a value of 0. Conversely, for factors negatively associated with survival (e.g., immune evasion [PC3]), low-scoring tumors were assigned a value of 1 and high-scoring tumors a value of 0. The TIME Score was then calculated as the sum of these binarized values, yielding a range from 0 to 5. Intuitively, this score represents the number of factors for a tumor that align with favorable prognosis, based on the univariate associations observed across the cohort (Figure 6A). As shown in Figure 6B, we find that the TIME Score monotonically stratifies patients by risk ($p = 4.3e-17$, p value adjusted for cancer type and stage). For each cancer type and stage, both high and low scores are observed, though certain cancer types (e.g., endometrial and RCC) have a greater proportion of high scores than others (e.g., pancreatic and breast) (Figure 6C). The prognostic significance of the score also varies by cancer type, with significant stratification observed for some cancers (breast, CRC, NSCLC, and esophagogastric) but not others (Figure 7), which may reflect the variability in the prognostic impact of the individual TIME factors per cancer type (Figure 6A).

DISCUSSION

We characterized tumor-immune topographic organization across a real-world, pan-cancer cohort of 2,019 patients. By combining density and spatial metrics of key immune biomarkers, we identified five TIME spatial factors that are conserved across cancer types and stages. The factors provide different axes to describe tumors in a data-driven fashion. We quantified trends in the distribution of the factors across clinicopathological variables, including identifying associations with molecular signatures and patient prognosis.

While some TIME spatial factors and their associations align with biological intuition and prior findings, unexpected trends also emerge. Descriptors of "hot/cold" and "excluded/mixed" are commonly used in immuno-oncology,[31] and these axes emerge as the first two factors in our unsupervised analysis. This analysis results in quantitative definitions of these commonly used terms and highlights other considerations, such as including high PD-L1 TPS as a component of the immune "hot" phenotype. Other intuitive factors such as the difference between cytotoxic $CD8^+$ T cell and regulatory $FOXP3^+$ T cell enrichment also emerge from our analysis. The immune evasion (PC3) factor provides a nuanced means to describe the TIME, wherein tumors scoring high for this factor have high PD-L1 TPS, high PD-$L1^+$ cell proximity to tumor cells, and low $CD8^+$ cell density. Studies have shown that PD-$L1^+$ immune cells are commonly macrophages[32] and certain tumor-associated macrophages can promote immune evasion.[17,33,34] Since the TIME spatial factors correspond to orthogonal sources of variation across the cohort, they can be used as complementary ways to describe tumors. For instance, a tumor could be hot but also score high for immune evasion based on the relative differences between certain immune biomarker densities and spatial arrangements.

Unsupervised decomposition performed on different case subsets showed results largely consistent with those obtained across the entire cohort, suggesting that the identified factors may represent fundamental properties of tumor-immune cell interactions that are conserved across solid tumors. Furthermore, cancer type and stage accounted for <20% of the variance in the distribution of factor scores across the cohort. Nonetheless, several trends were observed. The factors that varied most by cancer type were immune enrichment (PC1) and Treg/cytotoxic enrichment (PC5). Head and neck cancer, NSCLC, and melanoma scored the highest for immune enrichment, aligning with the general understanding that these are hotter tumors.[31,35] In contrast, the coldest tumors were breast, prostate, and pancreatic, also aligning with prior knowledge.[31,35] In terms of the relative difference between Treg and cytotoxic T cell enrichment, head and neck cancer was the most Treg enriched, aligning with previous reports of a high FOXP3/CD8 ratio in this cancer type,[36,37] and RCC was the most cytotoxic T cell enriched.

Figure 5. Associations between TIME spatial factors and copy number alterations

(A) Counts of genes with a copy number alteration (CNA) gain/amplification.

(B) Van Elteren T-statistic per gene and TIME spatial factor. Genes with a significant association with at least one factor are displayed based on a Benjamini-Hochberg correction with a false discovery rate of 10%. Genes are ordered by chromosome arm.

(C) Counts of genes with a CNA loss/deletion.

(D) Van Elteren T-statistic per gene and TIME spatial factor.

$^*p < 0.1$, $^{**}p < 0.01$, and $^{***}p < 0.001$.

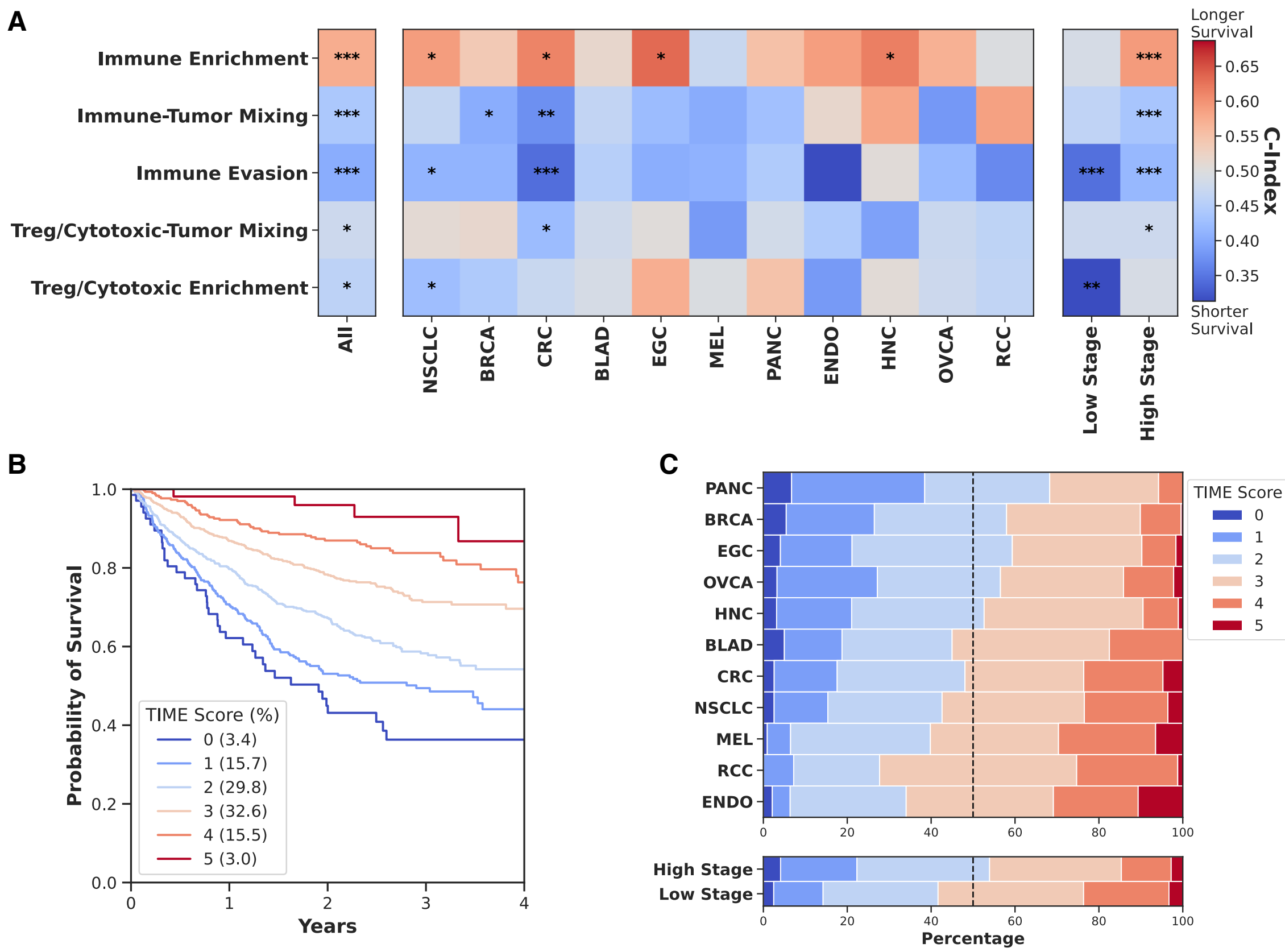

Figure 6. Associations between TIME spatial factors and patient prognosis

(A) Concordance index (C-index) between factors and patient overall survival for the entire cohort, per cancer type, and per stage. Values above 0.50 indicate positive correlation with survival, values below 0.50 indicate negative correlation with survival, and a value of 0.50 indicates a chance result. Cancer types with at least 80 cases are displayed.

(B) Kaplan-Meier curve based on the TIME Score across the cohort. The TIME Score represents the number of factors that are in the positive direction for survival for a given case.

(C) Distribution of TIME Score by cancer type and stage. Subgroups are sorted according to the average score. Low and high scores are present for each subset. NSCLC, non-small cell lung cancer; BRCA, breast cancer; CRC, colorectal cancer; BLAD, bladder cancer; EGC, esophagogastric cancer; MEL, melanoma; PANC, pancreatic cancer; HNC, head and neck cancer; ENDO, endometrial cancer; OVCA, ovarian cancer; RCC, renal cell carcinoma.

**p* < 0.1, ***p* < 0.01, and ****p* < 0.001.

In terms of genomic alterations, the largest number of associations was observed for the immune enrichment (PC1) factor. Somatic mutations in genes involved in the DNA mismatch repair (MMR) pathway—*MLH3*, *MSH6*, and *PMS2*—were associated with higher immune enrichment scores, consistent with the high immunogenicity of MMR-deficient tumors. Beta2-microglobulin (*B2M*) also exhibited a strong positive association with immune enrichment. *B2M* alterations have been shown to correlate with poor immunotherapy response,[38,39] but are also associated with MMR deficiency and high TMB.[39] *EGFR* mutant tumors demonstrated the strongest negative association with immune enrichment, aligning with evidence of an uninflamed phenotype.[40] Notably, lung adenocarcinomas with activating *EGFR* mutations are typically unresponsive to immunotherapy.[41] Additionally, *APC* mutations were negatively associated with the immune-tumor mixing (PC2) factor, suggesting an immune-excluded phenotype. A prior study identified a negative correlation between *APC* mutation and immunotherapy response in CRC,[42] so our results may further help explain this finding. *TP53* mutations were positively associated with immune evasion (PC3) and Treg/cytotoxic enrichment (PC5), consistent with studies linking *TP53* with PD-L1 and FOXP3 expression.[43–46] Finally, *ATRX* mutations were negatively associated with the Treg/cytotoxic-tumor mixing (PC4) factor, indicating a correlation with higher cytotoxic T cell proximity to tumor cells relative to Treg proximity. This finding may align with a prior study reporting increased immunotherapy efficacy and increased T cell infiltration in *ATRX*-mutant NSCLC tumors.[47]

For CNAs, positive associations were found between gains in chromosome 9p24.1 genes—*CD274* (PD-L1), *JAK2*, and *PDCD1LG2* (PD-L2)—and both immune enrichment (PC1) and immune evasion (PC3). 9p24.1 amplification has been previously associated with high, but ineffective immune infiltration with high PD-L1 expression in classical Hodgkin's lymphoma.[48] Our findings suggest that these associations may be more widespread. These findings also align with evidence that 9p24.1 losses are

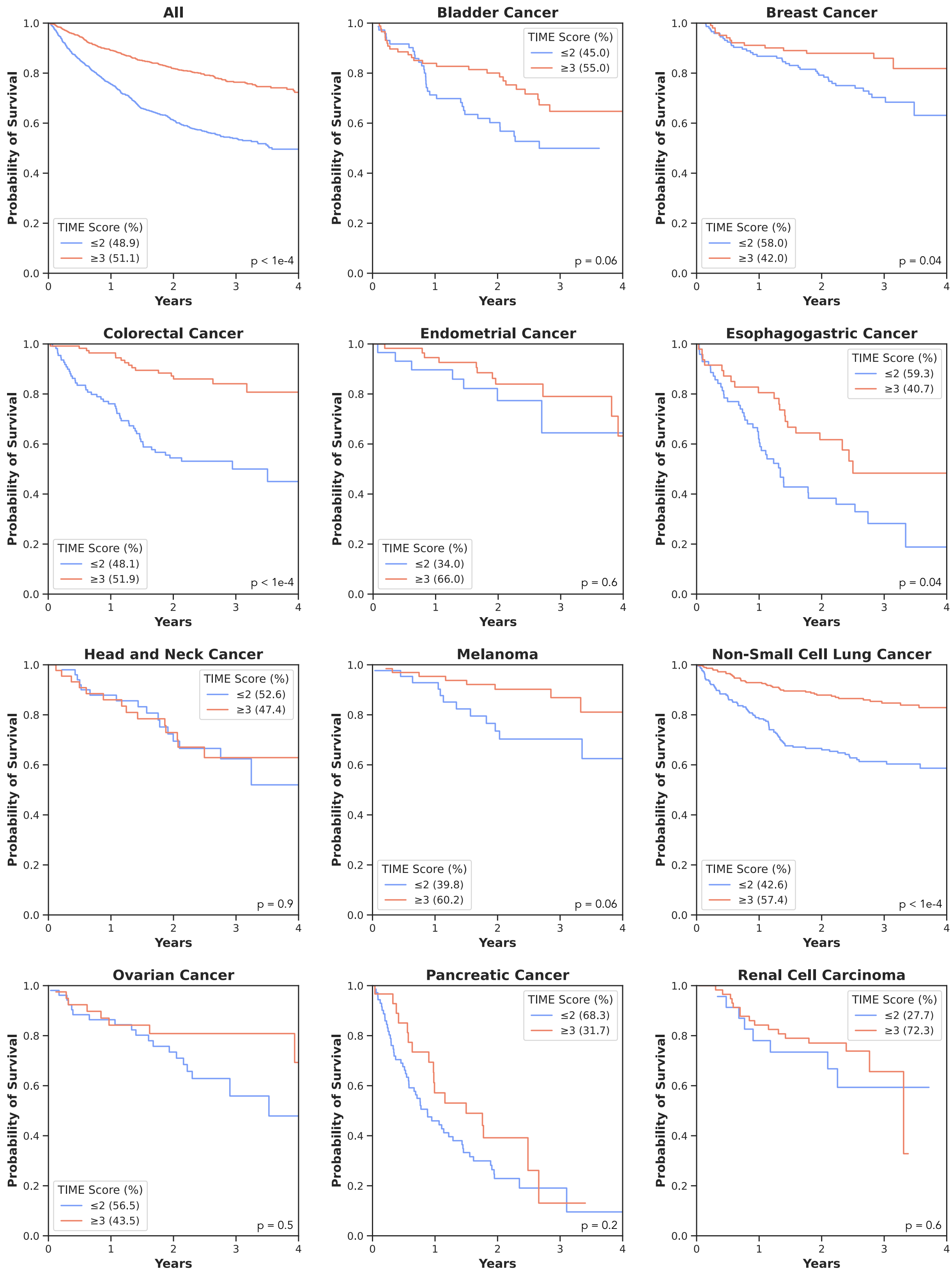

(legend on next page)

linked to immune-cold, immune checkpoint inhibitor (ICI)-resistant tumors, while 9p24.1 gains are associated with immune-hot, ICI-responsive tumors in head and neck cancer.[49] In addition, 9p arm-level loss, which includes *CDKN2A/B* and *MTAP*, was previously associated with impaired outcomes to immunotherapy in advanced NSCLC.[50] Of all genetic alterations and TIME spatial factors, the strongest associations were found between amplification of 20q13.2 genes *BCL2L1*, *AURKA*, and *ZNF217* and decreased immune enrichment (PC1). Aurora-A (AURKA) inhibitors are an emerging targeted therapy, and studies suggest that their use can enhance immune infiltration.[51,52] Finally, activating mutations of *PIK3CA* have been correlated with PD-L1 expression[53] in CRC and improved survival[54] for CRC patients treated with celecoxib (COX2-targeted anti-inflammatory), and gains in 3q genes have been associated with ICI response in cutaneous squamous cell carcinoma.[55] In our cohort, gains in 3q genes *PIK3CA* and *BCL6* were negatively associated with immune enrichment (PC1) and positively associated with immune evasion (PC3). Overall, we note that it is difficult to isolate gene-level effects from what may be larger region or arm-level alterations with CNA analysis, but our findings nonetheless provide insights into the association between the TIME and genomic phenotypes. Furthermore, we stratified by cancer type when assessing genomic associations and only included alterations that are present in at least five cancer types, but some associations may still be driven by skewed occurrence in specific cancers (Figures 4A, 5A, and 5C).

Although our primary aim was to characterize TIME variation and its biological correlates, each TIME spatial factor was associated with patient OS. The most prognostic factor was immune evasion (PC3), which showed consistently negative correlations with survival across cancer types and stages. Immune enrichment (PC1) and Treg/cytotoxic enrichment (PC5) were positively and negatively correlated with survival, respectively; however, these trends varied by stage. The immune enrichment pattern was driven by high stage cases, whereas the Treg/cytotoxic enrichment pattern was driven by low stage cases. This may suggest that the extent of immune infiltration is prognostically important as cancer progresses, but the relative type of infiltration is most important at an early stage. For the immune-tumor mixing (PC2) factor, an unexpected negative association with survival was observed. There may be several reasons why this is the case. First, this factor has positive weights for the proximity scores of all the immune biomarkers, whereas the association with survival may be biomarker specific. Indeed, relatively higher cytotoxic T cell proximity than Treg proximity to tumor cells was positively correlated with survival, as quantified by the Treg/cytotoxic-tumor mixing (PC4) factor. Second, while the immune-tumor mixing (PC2) factor had the highest weights for the proximity scores, slightly negative weights were also present for the cell density metrics, which may also contribute to the negative correlation with prognosis. A composite score combining all factors significantly stratified survival across the cohort, though stratification varied by cancer type. While different cutoff thresholds may improve separation for certain cancers, this variability likely stems from differences in the prognostic value of the individual factors comprising the TIME score. For example, the immune enrichment (PC1) factor was prognostic for some cancer types but not others, as measured by the C-index that quantifies prognostication independent of score cutoffs. Altogether, the prognosis analysis highlights the potential of quantitative, image-based prognostic biomarkers, but future work is necessary to maximize this prognostic accuracy, such as using supervised machine learning techniques. For instance, our group has shown that $CD8^+$ cell density alone is significantly prognostic in the ImmunoProfile cohort,[19,20] demonstrating similar prognostic accuracy (C-index of 0.61) as the immune evasion factor (C-index of 0.60) identified in this study, which weighs $CD8^+$ cell density and PD-L1 metrics in an unsupervised fashion.

In contrast to prior pan-cancer tumor subtyping analyses using cohorts such as TCGA[56,57] and CPTAC,[58] our analysis captures single-cell, spatial aspects of the TIME through a prospectively performed mIF assay. Given differences in methodological approaches and aspects of the TIME being measured, it is challenging to directly compare our TIME spatial factors to these previously reported immune subtypes. The most relevant comparison arguably lies in the existence of hot versus cold descriptors across the different schemes, such as the immune enrichment (PC1) factor in our work, inflammatory and lymphocyte-depleted subtypes in Thorsson et al.,[56] immune-enriched and immune-depleted subtypes in Bagaev et al.,[57] and the $CD8^+/IFNG^+$ and $CD8^-/IFNG^-$ subtypes in Petralia et al.[58]

Limitations of the study

While large-scale, pan-cancer mIF studies are currently lacking, higher-plexed assays have been performed in smaller studies that image tens of biomarkers compared to five in the ImmunoProfile cohort. Thus, our analysis is inherently confined to the studied biomarkers; however, these biomarkers (i.e., CD8, FOXP3, PD-1, and PD-L1) have been implicated in many aspects of tumor immunity.[59–61] Furthermore, the focused panel size enhances clinical scalability, enabling us to investigate tumor, genomic, and clinical associations across a cohort of 2,019 patients—yielding insights that are not feasible to study in smaller cohorts with higher-plex assays. An additional challenge, but also a strength, is the heterogeneity of the studied cohort. Representing an unselected clinical population, the cohort consists of patients undergoing varying treatment regimens across different cancer types. Nonetheless, our core findings were robust across cancer types and stages, enabling the identification of overarching associations as well as cancer specific trends. The prognosis analysis should especially be interpreted in the context of the current standard of care, where treatment-specific effects are challenging to estimate in pan-cancer settings and may shift as new approaches emerge. Additionally,

Figure 7. Kaplan-Meier curves by TIME Score per cancer type

The TIME Score is formulated based on the entire cohort and is displayed per cancer type. Scores are split into low (≤ 2) or high (≥ 3) due to sample sizes. Two-sided p values are computed via the log rank test and are Benjamini-Hochberg corrected for multiple comparisons.

our methodological approach was designed to disentangle immune biomarker densities from their tumor proximity and result in interpretable factors, but higher-order levels of spatial organization would also be useful to analyze, including non-intratumoral regions, intratumoral heterogeneity, and WSI analysis. The ROI sampling strategy utilized here leverages pathologist expertise and reduces computational costs, but WSI-level analysis or automated ROI sampling strategies may ultimately reduce sampling biases and enhance reproducibility across clinical sites. Additionally, our analysis involves the averaging of metrics (cell densities and spatial proximities) across ROIs for a given sample, leaving opportunities to study intratumoral heterogeneity. Spatial proximity scores between different immune cell types could also be considered, as our analysis focused on tumor-immune cell proximity. However, these scores would be subject to high noise levels given the lower abundance of immune cells than tumor cells in most samples, which becomes compounded when analyzing pairs of distinct immune cell types.

Understanding the spatial organization of the TIME and its relation to genomic phenotypes and patient outcomes is a critical path forward in precision oncology. Our analysis and accompanying database of 39.4 million spatially resolved cells are designed to accelerate progress in this direction.

RESOURCE AVAILABILITY

Lead contact

Further information and requests for resources and reagents should be directed to and will be fulfilled by the lead contact, William Lotter (lotterb@ds.dfci.harvard.edu).

Materials availability

This study did not generate new unique reagents.

Data and code availability

- The de-identified single-cell database with biomarker expression, genomic, and clinical annotations has been deposited at Synapse and is publicly available at https://doi.org/10.7303/syn52596661 as of the date of publication.
- All original code has been deposited on GitHub and is publicly available at https://github.com/lotterlab/time_spatial_factors as of the date of publication.
- Any additional information required to reanalyze the data reported in this work is available from the lead contact upon request.

ACKNOWLEDGMENTS

The authors thank the Dana-Farber Cancer Institute and its patients and donors, whose charitable contributions helped support this study. W.L. gratefully acknowledges funding support from the Ellison Foundation, the Wong Family Award in Translational Oncology, the National Institute of Biomedical Imaging and Bioengineering award R21EB035247, and the National Library of Medicine award R01LM014775.

AUTHOR CONTRIBUTIONS

Conceptualization, J.R.L., J.A., J.V.A., F.S.H., N.L., L.M.S., E.C., J.A.N., M.M.A., S.J.R., and W.L.; data curation, J.R.L., J.A., J.L.W., I.D., B.S., K.F., S.J.R., and W.L.; formal analysis, J.R.L., J.A., J.V.A., A.G.-H., and W.L.; writing – original draft, J.R.L., J.A., and W.L.; writing – review & editing, all authors.

DECLARATION OF INTERESTS

J.V.A. is an advisory board member for BMS, AstraZeneca, Janssen, and Amgen and reports speaker activities for MSD, Janssen, AstraZeneca, and BMS. F.S.H. reports grants and personal fees from Bristol-Myers Squibb and Novartis and personal fees from Merck, Compass Therapeutics, Apricity, 7 Hills Pharma, Bicara, Checkpoint Therapeutics, Genentech/Roche, Bioentre, Gossamer, Iovance, Catalym, Immunocore, Kairos, Rheos, Bayer, Zumutor, Corner Therapeutics, Puretech, Curis, AstraZeneca, Pliant, Solu Therapeutics, Vir Biotechnology, and 92Bio, outside the submitted work. In addition, F.S.H. has a patent "Methods for treating MICA-related disorders" (no. 20100111973) with royalties paid, a patent "Tumor antigens and uses thereof" (no. 7250291; issued), a patent "Angiopoiten-2 biomarkers predictive of anti-immune checkpoint response" (no. 20170248603; pending), a patent "Compositions and methods for identification, assessment, prevention, and treatment of melanoma using PD-L1 isoforms" (no. 20160340407; pending), a patent "Therapeutic peptides" (no. 20160046716; pending), a patent "Therapeutic peptides" (no. 20140004112; pending), a patent "Therapeutic peptides" (no. 20170022275; pending), a patent "Therapeutic peptides" (no. 20170008962; pending), a patent "Therapeutic peptides" (no. 9402905; issued), a patent "Methods of using pembrolizumab and trebananib" (pending), a patent "Vaccine compositions and methods for restoring NKG2D pathway function against cancers" (no. 10279021) with royalties paid, a patent "Antibodies that bind to MHC class I polypeptide-related sequence A" (no. 10106611; issued), a patent "Anti-galectin antibody biomarkers predictive of anti-immune checkpoint and anti-angiogenesis responses" (no. 20170343552; pending), and a patent "Antibodies against EDIL3 and methods of use thereof" (pending). L.M.S. receives research support from Bristol Myers Squibb and Genentech and has received consulting income from Genentech, Lilly, and AstraZeneca. J.A.N. serves as a consult for Leica Biosystems and receives research support from Natera. S.J.R. receives research support from Bristol Myers Squibb, KITE/Gilead, and Surface Oncology and is an SAB member for Immunitas Therapeutics

STAR★METHODS

Detailed methods are provided in the online version of this paper and include the following:

- KEY RESOURCES TABLE
- EXPERIMENTAL MODEL AND STUDY PARTICIPANT DETAILS
  - Ethics statement
  - Clinical cohort
- METHOD DETAILS
  - ImmunoProfile assay
  - Regions of interest selection
  - Cell segmentation
  - Image analysis reproducibility studies
  - Implementation of batch controls
  - Proximity score metric
  - Principal component analysis
  - Associations with cancer type and stage
  - Associations with genomic alterations
  - Associations with patient prognosis
- QUANTIFICATION AND STATISTICAL ANALYSIS

SUPPLEMENTAL INFORMATION

Supplemental information can be found online at https://doi.org/10.1016/j.xcrm.2025.102418.

Received: February 24, 2025
Revised: June 27, 2025
Accepted: September 22, 2025
Published: October 14, 2025

REFERENCES

1. de Souza, N., Zhao, S., and Bodenmiller, B. (2024). Multiplex protein imaging in tumour biology. Nat. Rev. Cancer *24*, 171–191. https://doi.org/10.1038/s41568-023-00657-4.
2. Walsh, L.A., and Quail, D.F. (2023). Decoding the tumor microenvironment with spatial technologies. Nat. Immunol. *24*, 1982–1993. https://doi.org/10.1038/s41590-023-01678-9.
3. Pagès, F., Berger, A., Camus, M., Sanchez-Cabo, F., Costes, A., Molidor, R., Mlecnik, B., Kirilovsky, A., Nilsson, M., Damotte, D., et al. (2005). Effector memory T cells, early metastasis, and survival in colorectal cancer. N. Engl. J. Med. *353*, 2654–2666. https://doi.org/10.1056/NEJMoa051424.
4. Galon, J., Costes, A., Sanchez-Cabo, F., Kirilovsky, A., Mlecnik, B., Lagorce-Pagès, C., Tosolini, M., Camus, M., Berger, A., Wind, P., et al. (2006). Type, density, and location of immune cells within human colorectal tumors predict clinical outcome. Science *313*, 1960–1964. https://doi.org/10.1126/science.1129139.
5. Mahmoud, S.M.A., Paish, E.C., Powe, D.G., Macmillan, R.D., Grainge, M. J., Lee, A.H.S., Ellis, I.O., and Green, A.R. (2011). Tumor-infiltrating CD8+ lymphocytes predict clinical outcome in breast cancer. J. Clin. Oncol. *29*, 1949–1955. https://doi.org/10.1200/JCO.2010.30.5037.
6. Pagès, F., Mlecnik, B., Marliot, F., Bindea, G., Ou, F.S., Bifulco, C., Lugli, A., Zlobec, I., Rau, T.T., Berger, M.D., et al. (2018). International validation of the consensus Immunoscore for the classification of colon cancer: a prognostic and accuracy study. Lancet *391*, 2128–2139. https://doi.org/10.1016/S0140-6736(18)30789-X.
7. Tumeh, P.C., Harview, C.L., Yearley, J.H., Shintaku, I.P., Taylor, E.J.M., Robert, L., Chmielowski, B., Spasic, M., Henry, G., Ciobanu, V., et al. (2014). PD-1 blockade induces responses by inhibiting adaptive immune resistance. Nature *515*, 568–571. https://doi.org/10.1038/nature13954.
8. Berry, S., Giraldo, N.A., Green, B.F., Cottrell, T.R., Stein, J.E., Engle, E.L., Xu, H., Ogurtsova, A., Roberts, C., Wang, D., et al. (2021). Analysis of multispectral imaging with the AstroPath platform informs efficacy of PD-1 blockade. Science *372*, eaba2609. https://doi.org/10.1126/science.aba2609.
9. Lin, J.-R., Chen, Y.A., Campton, D., Cooper, J., Coy, S., Yapp, C., Tefft, J. B., McCarty, E., Ligon, K.L., Rodig, S.J., et al. (2023). High-plex immunofluorescence imaging and traditional histology of the same tissue section for discovering image-based biomarkers. Nat. Cancer *4*, 1036–1052. https://doi.org/10.1038/s43018-023-00576-1.
10. Sorin, M., Rezanejad, M., Karimi, E., Fiset, B., Desharnais, L., Perus, L.J. M., Milette, S., Yu, M.W., Maritan, S.M., Doré, S., et al. (2023). Single-cell spatial landscapes of the lung tumour immune microenvironment. Nature *614*, 548–554. https://doi.org/10.1038/s41586-022-05672-3.
11. Angelo, M., Bendall, S.C., Finck, R., Hale, M.B., Hitzman, C., Borowsky, A. D., Levenson, R.M., Lowe, J.B., Liu, S.D., Zhao, S., et al. (2014). Multiplexed ion beam imaging of human breast tumors. Nat. Med. *20*, 436–442. https://doi.org/10.1038/nm.3488.
12. Jackson, H.W., Fischer, J.R., Zanotelli, V.R.T., Ali, H.R., Mechera, R., Soysal, S.D., Moch, H., Muenst, S., Varga, Z., Weber, W.P., and Bodenmiller, B. (2020). The single-cell pathology landscape of breast cancer. Nature *578*, 615–620. https://doi.org/10.1038/s41586-019-1876-x.
13. Mo, C.-K., Liu, J., Chen, S., Storrs, E., Targino da Costa, A.L.N., Houston, A., Wendl, M.C., Jayasinghe, R.G., Iglesia, M.D., Ma, C., et al. (2024). Tumour evolution and microenvironment interactions in 2D and 3D space. Nature *634*, 1178–1186. https://doi.org/10.1038/s41586-024-08087-4.
14. Keren, L., Bosse, M., Marquez, D., Angoshtari, R., Jain, S., Varma, S., Yang, S.R., Kurian, A., Van Valen, D., West, R., et al. (2018). A Structured Tumor-Immune Microenvironment in Triple Negative Breast Cancer Revealed by Multiplexed Ion Beam Imaging. Cell *174*, 1373–1387.e19. https://doi.org/10.1016/j.cell.2018.08.039.
15. Wang, X.Q., Danenberg, E., Huang, C.S., Egle, D., Callari, M., Bermejo, B., Dugo, M., Zamagni, C., Thill, M., Anton, A., et al. (2023). Spatial predictors of immunotherapy response in triple-negative breast cancer. Nature *621*, 868–876. https://doi.org/10.1038/s41586-023-06498-3.
16. Parra, E.R., Zhang, J., Jiang, M., Tamegnon, A., Pandurengan, R.K., Behrens, C., Solis, L., Haymaker, C., Heymach, J.V., Moran, C., et al. (2023). Immune cellular patterns of distribution affect outcomes of patients with non-small cell lung cancer. Nat. Commun. *14*, 2364. https://doi.org/10.1038/s41467-023-37905-y.
17. Peng, H., Wu, X., Zhong, R., Yu, T., Cai, X., Liu, J., Wen, Y., Ao, Y., Chen, J., Li, Y., et al. (2021). Profiling tumor immune microenvironment of non-small cell lung cancer using multiplex immunofluorescence. Front. Immunol. *12*, 750046. https://doi.org/10.3389/fimmu.2021.750046.
18. Frei, A.L., McGuigan, A., Sinha, R.R.A.K., Jabbar, F., Gneo, L., Tomasevic, T., Harkin, A., Iveson, T., Saunders, M.P., Oien, K.A., et al. (2024). Multiplex analysis of intratumoural immune infiltrate and prognosis in patients with stage II-III colorectal cancer from the SCOT and QUASAR 2 trials: a retrospective analysis. Lancet Oncol. *25*, 198–211. https://doi.org/10.1016/S1470-2045(23)00560-0.
19. Lindsay, J., Sharma, B., Felt, K.D., Giobbie-Hurder, A., Dryg, I., Weirather, J.L., Altreuter, J., Mazor, T., Kumari, P., Alessi, J.V., et al. (2023). Immuno-PROFILE: A prospective implementation of clinically validated, quantitative immune cell profiling test identifies tumor-infiltrating CD8+ and PD-1 + cell densities as prognostic biomarkers across a 2,023 patient pan-cancer cohort treated with different therapies. Cancer Res. *83*, 5706. https://doi.org/10.1158/1538-7445.AM2023-5706.
20. Alessi, J.V., Lindsay, J.R., Giobbie-Hurder, A., Sharma, B., Felt, K., Kumari, P., Mazor, T., Cerami, E., Lotter, W., Altreuter, J., et al. (2025). Immunoprofiling at an Institutional Scale Reveals That High Numbers of Intratumoral CD8+ and PD-1+ Cells Predict Superior Patient Survival Across Major Cancer Types Independent of Major Risk Factors. JCO Precis. Oncol. *9*, e2500240. https://doi.org/10.1200/PO-25-00240.
21. Kundra, R., Zhang, H., Sheridan, R., Sirintrapun, S.J., Wang, A., Ochoa, A., Wilson, M., Gross, B., Sun, Y., Madupuri, R., et al. (2021). OncoTree: A cancer classification system for precision oncology. JCO Clin. Cancer Inform. *5*, 221–230. https://doi.org/10.1200/CCI.20.00108.
22. Garcia, E.P., Minkovsky, A., Jia, Y., Ducar, M.D., Shivdasani, P., Gong, X., Ligon, A.H., Sholl, L.M., Kuo, F.C., MacConaill, L.E., et al. (2017). Validation of OncoPanel: A Targeted Next-Generation Sequencing Assay for the Detection of Somatic Variants in Cancer. Arch. Pathol. Lab Med. *141*, 751–758. https://doi.org/10.5858/arpa.2016-0527-OA.
23. Sholl, L.M., Do, K., Shivdasani, P., Cerami, E., Dubuc, A.M., Kuo, F.C., Garcia, E.P., Jia, Y., Davineni, P., Abo, R.P., et al. (2016). Institutional implementation of clinical tumor profiling on an unselected cancer population. JCI Insight *1*, e87062. https://doi.org/10.1172/jci.insight.87062.
24. Van Lieshout, M.N.M., and Baddeley, A.J. (1999). Indices of Dependence between Types in Multivariate Point Patterns. Scandinavian J. Statistics *26*, 511–532. https://doi.org/10.1111/1467-9469.00165.
25. Parra, E.R. (2021). Methods to Determine and Analyze the Cellular Spatial Distribution Extracted From Multiplex Immunofluorescence Data to Understand the Tumor Microenvironment. Front. Mol. Biosci. *8*, 668340. https://doi.org/10.3389/fmolb.2021.668340.
26. Page, D.B., Broeckx, G., Jahangir, C.A., Verbandt, S., Gupta, R.R., Thagaard, J., Khiroya, R., Kos, Z., Abduljabbar, K., Acosta Haab, G., et al. (2023). Spatial analyses of immune cell infiltration in cancer: current methods and future directions: A report of the International Immuno-Oncology Biomarker Working Group on Breast Cancer. J. Pathol. *260*, 514–532. https://doi.org/10.1002/path.6165.
27. Hoffman, G.E., and Schadt, E.E. (2016). variancePartition: interpreting drivers of variation in complex gene expression studies. BMC Bioinf. *17*, 483. https://doi.org/10.1186/s12859-016-1323-z.
28. Benjamini, Y., and Hochberg, Y. (1995). Controlling the false discovery rate: A practical and powerful approach to multiple testing. J. R. Stat. Soc. Series B Stat. Methodol. *57*, 289–300. https://doi.org/10.1111/j.2517-6161.1995.tb02031.x.

29. Chakravarty, D., Gao, J., Phillips, S.M., Kundra, R., Zhang, H., Wang, J., Rudolph, J.E., Yaeger, R., Soumerai, T., Nissan, M.H., et al. (2017). OncoKB: A precision oncology knowledge base. JCO Precis. Oncol. *2017*, PO.17.00011. https://doi.org/10.1200/PO.17.00011.

30. Suehnholz, S.P., Nissan, M.H., Zhang, H., Kundra, R., Nandakumar, S., Lu, C., Carrero, S., Dhaneshwar, A., Fernandez, N., Xu, B.W., et al. (2024). Quantifying the expanding landscape of clinical actionability for patients with cancer. Cancer Discov. *14*, 49–65. https://doi.org/10.1158/2159-8290.CD-23-0467.

31. Galon, J., and Bruni, D. (2019). Approaches to treat immune hot, altered and cold tumours with combination immunotherapies. Nat. Rev. Drug Discov. *18*, 197–218. https://doi.org/10.1038/s41573-018-0007-y.

32. Liu, Y., Zugazagoitia, J., Ahmed, F.S., Henick, B.S., Gettinger, S.N., Herbst, R.S., Schalper, K.A., and Rimm, D.L. (2020). Immune cell PD-L1 colocalizes with macrophages and is associated with outcome in PD-1 pathway blockade therapy. Clin. Cancer Res. *26*, 970–977. https://doi.org/10.1158/1078-0432.CCR-19-1040.

33. Zheng, X., Weigert, A., Reu, S., Guenther, S., Mansouri, S., Bassaly, B., Gattenlöhner, S., Grimminger, F., Pullamsetti, S., Seeger, W., et al. (2020). Spatial density and distribution of tumor-associated macrophages predict survival in non-small cell lung carcinoma. Cancer Res. *80*, 4414–4425. https://doi.org/10.1158/0008-5472.CAN-20-0069.

34. Carey, C.D., Gusenleitner, D., Lipschitz, M., Roemer, M.G.M., Stack, E.C., Gjini, E., Hu, X., Redd, R., Freeman, G.J., Neuberg, D., et al. (2017). Topological analysis reveals a PD-L1-associated microenvironmental niche for Reed-Sternberg cells in Hodgkin lymphoma. Blood *130*, 2420–2430. https://doi.org/10.1182/blood-2017-03-770719.

35. Binnewies, M., Roberts, E.W., Kersten, K., Chan, V., Fearon, D.F., Merad, M., Coussens, L.M., Gabrilovich, D.I., Ostrand-Rosenberg, S., Hedrick, C. C., et al. (2018). Understanding the tumor immune microenvironment (TIME) for effective therapy. Nat. Med. *24*, 541–550. https://doi.org/10.1002/med.21765.

36. Mandal, R., Şenbabaoğlu, Y., Desrichard, A., Havel, J.J., Dalin, M.G., Riaz, N., Lee, K.W., Ganly, I., Hakimi, A.A., Chan, T.A., and Morris, L.G. (2016). The head and neck cancer immune landscape and its immunotherapeutic implications. JCI Insight *1*, e89829. https://doi.org/10.1172/jci.insight.89829.

37. Seminerio, I., Descamps, G., Dupont, S., de Marrez, L., Laigle, J.A., Lechien, J.R., Kindt, N., Journe, F., and Saussez, S. (2019). Infiltration of FoxP3+ Regulatory T Cells is a Strong and Independent Prognostic Factor in Head and Neck Squamous Cell Carcinoma. Cancers *11*, 227. https://doi.org/10.3390/cancers11020227.

38. Zaretsky, J.M., Garcia-Diaz, A., Shin, D.S., Escuin-Ordinas, H., Hugo, W., Hu-Lieskovan, S., Torrejon, D.Y., Abril-Rodriguez, G., Sandoval, S., Barthly, L., et al. (2016). Mutations associated with acquired resistance to PD-1 blockade in melanoma. N. Engl. J. Med. *375*, 819–829. https://doi.org/10.1056/NEJMoa1604958.

39. Wang, H., Liu, B., and Wei, J. (2021). Beta2-microglobulin(B2M) in cancer immunotherapies: Biological function, resistance and remedy. Cancer Lett. *517*, 96–104. https://doi.org/10.1016/j.canlet.2021.06.008.

40. Dong, Z.-Y., Zhang, J.T., Liu, S.Y., Su, J., Zhang, C., Xie, Z., Zhou, Q., Tu, H.Y., Xu, C.R., Yan, L.X., et al. (2017). EGFR mutation correlates with uninflamed phenotype and weak immunogenicity, causing impaired response to PD-1 blockade in non-small cell lung cancer. OncoImmunology *6*, e1356145. https://doi.org/10.1080/2162402X.2017.1356145.

41. Mazieres, J., Drilon, A., Lusque, A., Mhanna, L., Cortot, A.B., Mezquita, L., Thai, A.A., Mascaux, C., Couraud, S., Veillon, R., et al. (2019). Immune checkpoint inhibitors for patients with advanced lung cancer and oncogenic driver alterations: results from the IMMUNOTARGET registry. Ann. Oncol. *30*, 1321–1328. https://doi.org/10.1093/annonc/mdz167.

42. Li, B., Zhang, G., and Xu, X. (2023). APC mutation correlated with poor response of immunotherapy in colon cancer. BMC Gastroenterol. *23*, 95. https://doi.org/10.1186/s12876-023-02725-3.

43. Kim, J.-E., Shin, J.S., Moon, J.H., Hong, S.W., Jung, D.J., Kim, J.H., Hwang, I.Y., Shin, Y.J., Gong, E.Y., Lee, D.H., et al. (2017). Foxp3 is a key downstream regulator of p53-mediated cellular senescence. Oncogene *36*, 219–230. https://doi.org/10.1038/onc.2016.193.

44. Kawashima, H., Takatori, H., Suzuki, K., Iwata, A., Yokota, M., Suto, A., Minamino, T., Hirose, K., and Nakajima, H. (2013). Tumor suppressor p53 inhibits systemic autoimmune diseases by inducing regulatory T cells. J. Immunol. *191*, 3614–3623. https://doi.org/10.4049/jimmunol.1300509.

45. Sun, H., Liu, S.Y., Zhou, J.Y., Xu, J.T., Zhang, H.K., Yan, H.H., Huan, J.J., Dai, P.P., Xu, C.R., Su, J., et al. (2020). Specific TP53 subtype as biomarker for immune checkpoint inhibitors in lung adenocarcinoma. EBioMedicine *60*, 102990. https://doi.org/10.1016/j.ebiom.2020.102990.

46. Efe, G., Rustgi, A.K., and Prives, C. (2024). p53 at the crossroads of tumor immunity. Nat. Cancer *5*, 983–995. https://doi.org/10.1038/s43018-024-00796-z.

47. Hou, T., Jiang, S., Wang, Y., Xie, Y., Zhang, H., Feng, Y., Ma, F., Ma, J., Liu, X., and Hu, C. (2020). Alpha thalassemia/intellectual disability X-linked deficiency sensitizes non-small cell lung cancer to immune checkpoint inhibitors. Front. Oncol. *10*, 608300. https://doi.org/10.3389/fonc.2020.608300.

48. Green, M.R., Monti, S., Rodig, S.J., Juszczynski, P., Currie, T., O'Donnell, E., Chapuy, B., Takeyama, K., Neuberg, D., Golub, T.R., et al. (2010). Integrative analysis reveals selective 9p24.1 amplification, increased PD-1 ligand expression, and further induction via JAK2 in nodular sclerosing Hodgkin lymphoma and primary mediastinal large B-cell lymphoma. Blood *116*, 3268–3277. https://doi.org/10.1182/blood-2010-05-282780.

49. Zhao, X., Cohen, E.E.W., William, W.N., Jr., Bianchi, J.J., Abraham, J.P., Magee, D., Spetzler, D.B., Gutkind, J.S., Alexandrov, L.B., Cavenee, W. K., et al. (2022). Somatic 9p24.1 alterations in HPV- head and neck squamous cancer dictate immune microenvironment and anti-PD-1 checkpoint inhibitor activity. Proc. Natl. Acad. Sci. USA *119*, e2213835119. https://doi.org/10.1073/pnas.2213835119.

50. Alessi, J.V., Wang, X., Elkrief, A., Ricciuti, B., Li, Y.Y., Gupta, H., Spurr, L. F., Rizvi, H., Luo, J., Pecci, F., et al. (2023). Impact of aneuploidy and chromosome 9p loss on tumor immune microenvironment and immune checkpoint inhibitor efficacy in NSCLC. J. Thorac. Oncol. *18*, 1524–1537. https://doi.org/10.1016/j.jtho.2023.05.019.

51. Han, J., Jiang, Z., Wang, C., Chen, X., Li, R., Sun, N., Liu, X., Wang, H., Hong, L., Zheng, K., et al. (2020). Inhibition of Aurora-A promotes CD8+ T-cell infiltration by mediating IL10 production in cancer cells. Mol. Cancer Res. *18*, 1589–1602. https://doi.org/10.1158/1541-7786.MCR-19-1226.

52. Zheng, D., Li, J., Yan, H., Zhang, G., Li, W., Chu, E., and Wei, N. (2023). Emerging roles of Aurora-A kinase in cancer therapy resistance. Acta Pharm. Sin. B *13*, 2826–2843. https://doi.org/10.1016/j.apsb.2023.03.013.

53. Ahn, A.R., Kim, K.M., Jang, K.Y., Moon, W.S., Ha, G.W., Lee, M.R., and Chung, M.J. (2021). Correlation of PIK3CA mutation with programmed death ligand-1 (PD-L1) expression and their clinicopathological significance in colorectal cancer. Ann. Transl. Med. *9*, 1406. https://doi.org/10.21037/atm-21-2315.

54. Nowak, J.A., Twombly, T., Ma, C., Shi, Q., Haruki, K., Fujiyoshi, K., Väyrynen, J., Zhao, M., Knight, J., Mane, S., et al. (2024). Improved survival with adjuvant cyclooxygenase 2 inhibition in PIK3CA-activated stage III colon cancer: CALGB/SWOG 80702 (alliance). J. Clin. Oncol. *42*, 2853–2859. https://doi.org/10.1200/JCO.23.01680.

55. Kacew, A.J., Harris, E.J., Lorch, J.H., Haddad, R.I., Chau, N.G., Rabinowits, G., LeBoeuf, N.R., Schmults, C.D., Thakuria, M., MacConaill, L.E., and Hanna, G.J. (2019). Chromosome 3q arm gain linked to immunotherapy response in advanced cutaneous squamous cell carcinoma. Eur. J. Cancer *113*, 1–9. https://doi.org/10.1016/j.ejca.2019.03.004.

56. Thorsson, V., Gibbs, D.L., Brown, S.D., Wolf, D., Bortone, D.S., Ou Yang, T.H., Porta-Pardo, E., Gao, G.F., Plaisier, C.L., Eddy, J.A., et al. (2018). The

CellPress
OPEN ACCESS

Immune Landscape of Cancer. Immunity *48*, 812–830.e14. https://doi.org/10.1016/j.immuni.2018.03.023.

57. Bagaev, A., Kotlov, N., Nomie, K., Svekolkin, V., Gafurov, A., Isaeva, O., Osokin, N., Kozlov, I., Frenkel, F., Gancharova, O., et al. (2021). Conserved pan-cancer microenvironment subtypes predict response to immunotherapy. Cancer Cell *39*, 845–865.e7. https://doi.org/10.1016/j.ccell.2021.04.014.

58. Petralia, F., Ma, W., Yaron, T.M., Caruso, F.P., Tignor, N., Wang, J.M., Charytonowicz, D., Johnson, J.L., Huntsman, E.M., Marino, G.B., et al. (2024). Pan-cancer proteogenomics characterization of tumor immunity. Cell *187*, 1255–1277.e27. https://doi.org/10.1016/j.cell.2024.01.027.

59. Bruni, D., Angell, H.K., and Galon, J. (2020). The immune contexture and Immunoscore in cancer prognosis and therapeutic efficacy. Nat. Rev. Cancer *20*, 662–680. https://doi.org/10.1038/s41568-020-0285-7.

60. Sharma, P., Goswami, S., Raychaudhuri, D., Siddiqui, B.A., Singh, P., Nagarajan, A., Liu, J., Subudhi, S.K., Poon, C., Gant, K.L., et al. (2023). Immune checkpoint therapy—current perspectives and future directions. Cell *186*, 1652–1669. https://doi.org/10.1016/j.cell.2023.03.006.

61. Togashi, Y., Shitara, K., and Nishikawa, H. (2019). Regulatory T cells in cancer immunosuppression - implications for anticancer therapy. Nat. Rev. Clin. Oncol. *16*, 356–371. https://doi.org/10.1038/s41571-019-0175-7.

62. Baddeley, A., Turner, R. & Rubak, E. Spatstat: Spatial point pattern analysis, model-fitting, simulation, tests. CRAN: Contributed Packages The R Foundation https://doi.org/10.32614/cran.package.spatstat (2002).

63. Van Elteren, P.H. (1960). On the combination of independent two-sample tests of Wilcoxon. Bulletin of the International Statistical Institute *37*, 351–361.

64. Kloke, J. & McKean, J. npsm: Nonparametric Statistical Methods. CRAN: Contributed Packages The R Foundation https://doi.org/10.32614/cran.package.npsm (2014).

65. Cox, D.R. (1972). Regression Models and Life-Tables. J. R. Stat. Soc. Series B Stat. Methodol. *34*, 187–202. https://doi.org/10.1111/j.2517-6161.1972.tb00899.x.

STAR★METHODS

KEY RESOURCES TABLE

REAGENT or RESOURCE	SOURCE	IDENTIFIER
Antibodies		
CD8 antibody	Leica	Cat# NCL-L-CD8-4B11
PD-L1 antibody	Cell Signaling	Cat# 13684
FOXP3 antibody	Cell Signaling	Cat# 12653
PD-1 antibody	Abcam	Cat# Ab137132
Cytokeratin antibody	Agilent	Cat# M351501-2
SOX10 antibody	Cell Marque	Cat# AC-0237
PAX8 antibody	ProteinTech	Cat# 10336-1-AP
Deposited data		
Single-cell database	This paper	https://doi.org/10.7303/syn52596661
Software and algorithms		
time_spatial_factors	This paper	https://github.com/lotterlab/time_spatial_factors
inForm 2.4.8	Akoya Biosciences	https://www.akoyabio.com/phenoimager/inform-tissue-finder/
OncoKB-Annotator	OncoKB	https://github.com/oncokb/oncokb-annotator
npsm 1.1.1	CRAN	https://doi.org/10.32614/cran.package.npsm
Pythologist	Dana-Farber Cancer Institute	https://github.com/dfci/pythologist
variancePartition 3.2.1	Bioconductor	https://doi.org/10.18129/B9.bioc.variancePartition
spatstat 3.3.0	CRAN	https://doi.org/10.32614/cran.package.spatstat

EXPERIMENTAL MODEL AND STUDY PARTICIPANT DETAILS

Ethics statement

The IRB of the DFCI provided ethical approval for this work. All materials were obtained from patients who had signed informed consent forms for the research use of their materials. This research was conducted under DFCI protocols 11–104, 17-000, 20-000, and 22–176.

Clinical cohort

Patients in the ImmunoProfile cohort were enrolled at the DFCI in Boston, MA between November 2018 to January 2022. Upon patient consent, the decision to order the ImmunoProfile assay was made by the patient's clinical team. All solid cancers besides sarcomas, pediatric tumors, and neuro-oncology cases were eligible for the assay. Data for each patient, including cancer type, vital status/date of death, systemic therapies received at DFCI, specimen date, and cancer stage, were curated from medical records and internal databases with a retrieval date of August 2022. To ensure accuracy, clinical domain experts manually reviewed each case against the patient's medical record, including confirming that the reported stage reflected the disease status at the time of specimen collection. From the original cohort of 2,023 cases, four cases were excluded from the analysis because of insufficient numbers of cells to reliably compute spatial metrics, resulting in the final number of 2,019 cases (each from a unique patient). The analyzed cohort is summarized in Table S1. As a real-world clinical cohort, patients underwent a variety of treatment regimens, including 466 (23%) patients having received immunotherapy. Of these 466 patients, 234 started immunotherapy after the date of the tissue specimen used for ImmunoProfile, 131 started immunotherapy before, and 101 had an unknown immunotherapy start date. A total of 631 (31%) patients experienced a death event, with a median of 326 days (IQR: [137, 587]) from tissue sample collection to death. For censored patients, the median follow-up time was 923 days (IQR: [684, 1164]). The mean age was 63.7 years (IQR: [56.2, 72.6]). The distribution of patient gender was 1213 (56%) female patients and 946 (44%) male patients. The distribution of self-reported race was as follows: 1838 (91.0%) white, 59 (2.9%) Asian, 58 (2.9%) Black, 30 (1.5%) Other, 4 (0.2%) Mixed, 1 (<0.1%) Native Hawaiian or Other Islander, and 29 (1.4%) Unknown/Declined.

METHOD DETAILS

ImmunoProfile assay

For each case, a formalin-fixed, paraffin-embedded (FFPE) tissue block from either a core needle biopsy or resection was sectioned to generate a hematoxylin and eosin (H&E) stained slide and an unstained slide. The H&E slide was digitized and reviewed by a

pathologist to confirm the diagnosis and assess tumor content. The unstained slide was used for automated mIF staining using a BOND Rx system (Leica Biosystems, Danvers, MA, USA). The mIF panel included five markers: CD8, FOXP3, PD-1, PD-L1, and a tumor-specific marker (cytokeratin for epithelial cancers, PAX8 for renal cell carcinomas, and SOX10 for melanomas). DAPI was used as a nuclear counterstain. The mIF-stained slides were imaged using a PhenoImager (Akoya Biosciences, Marlborough, MA, USA) scanner at 20× magnification.

ROIs within each mIF WSI were selected by a technician scientist and approved by a pathologist following a pre-defined SOP. ROIs were selected to be representative of the overall tumor microenvironment, with a target of 6 ROIs per slide (average 5.5) depending on the amount of available tissue (see "ROI Selection" for further details). Following ROI selection, the inForm software (version 2.4.8; Akoya Biosciences, Marlborough, MA, USA) was used to perform spectral unmixing, cell segmentation, and quantification of marker intensity for each cell following a pre-defined SOP. Spectral unmixing was performed using the autofluorescence channel. InForm's built-in cell segmentation algorithm was run with predefined parameter settings, followed by review and refinement by the scientist, and final approval by a pathologist (see "Cell Segmentation" for further details). The scientist then selected cells that were positive and negative for each biomarker in the ROI based on their assessment of staining patterns, which were used to train a random forest algorithm in the inForm software. The trained algorithm would then output positive and negative biomarker labels for the remainder of the cells in the ROI. These predictions were reviewed, and the algorithm was retrained as necessary, such as by selecting more cells to be included as training data. The scientist also annotated the tumor border if present for each ROI, specifically segmenting intratumor, tumor stromal interface, and stroma regions using an internally developed GNU Image Manipulation Program. This segmentation was performed manually based on visual inspection, where intratumor was defined as any region that is entirely composed of tumor, and tumor stromal interface was defined as the region where the tumor meets the normal tissue. Of the 2,019 cases, 1,091 (54%) had at least one ROI with the tumor stromal interface present. Based on the segmentation maps, custom algorithms were employed to calculate the PD-L1 TPS and the densities of non-tumor cells positive for each immune biomarker across the union of intratumor regions for a given case. The annotation tools and algorithms can be found at: https://github.com/dfci/pythologist. All of these steps and the subsequent results were reviewed and approved by a pathologist using a digital sign-out tool.

The ImmunoProfile assay and its workflow were validated within the CLIA-certified molecular diagnostics laboratory at the Brigham & Women's Hospital in Boston (CLIA license #22D2156930). Quality control testing resulted in the assay's full clinical validation in compliance with CLIA standards. This validation included demonstrating high concordance between the assay and IHC and consistent inter-run reproducibility over time.[20]

Regions of interest selection

The technician scientist was instructed to target 6 ROIs if possible, consisting of 3 intratumor and 3 with tumor stromal interface (if present) where intratumor was defined as any region that is entirely composed of tumor and tumor stromal interface was defined as the region where the tumor meets the normal tissue. The overall criterion for ROI selection was to be representative of the overall tumor microenvironment and its immune infiltration, including encompassing heterogeneity if present (i.e., by selecting a set of regions that represents this heterogeneity). More than 6 ROIs could be selected if necessary to capture this heterogeneity. Areas with tissue necrosis, bubbles, or staining artifacts were instructed to not be selected. All ROIs had a fixed rectangular shape of 925μm × 693μm, which corresponded to one 20× field of view in the inForm software. If the amount of tissue present was insufficient to select 6 ROIs, the technician selected as many as possible. The average number of ROIs per case was 5.5 (median: 6, IQR: [6, 6], range: 1–9). The total number of ROIs analyzed was 11,182 (29.8% containing tumor stromal interface). Of the 2,019 cases, 1,091 (54%) had at least one ROI with the tumor stromal interface present. The ROI selection for each case was reviewed and approved by a pathologist as being appropriately representative of the tissue section.

Cell segmentation

Cell segmentation was performed with the built-in inForm algorithm using DAPI for the nucleus component and cytokeratin and CD8 (if deemed present in the image) as membrane components to assist with nuclear splitting. The default settings used for the algorithm are displayed in Figure S4. Each case started with these settings, which were then refined by the technician scientist as necessary to achieve sufficient segmentation. This human-in-the-loop process was designed to address the heterogeneity in the cohort, rather than strictly assume that the same settings would be sufficient for every case. When making adjustments, the "Assisting component splitting" parameter was first prioritized in order to increase or decrease the splitting sensitivity. The optimized settings were then applied across the ROIs in the case. The resulting cell segmentation was reviewed and approved by a pathologist. The centroid of each segmented cell was used to assign its x-y coordinates in the ROI.

Image analysis reproducibility studies

In addition to the pathologist review and approval of ROI selection, cell segmentation, and biomarker quantification steps for each case, reproducibility studies were performed to support the robustness of the image analysis process. Twenty cases were selected and analyzed separately by two scientists using the defined process, followed by comparison of the quantified biomarker metrics. This experiment was performed under two conditions: once using the same pre-selected ROIs that were previously approved by pathologists and once using independently selected ROIs by the scientists. The results of this analysis are summarized in Table S2,

which reports the inter-observer R^2 between the quantified biomarker metrics (intratumor densities of CD8, PD-1, FOXP3 and PD-L1 TPS and PD-L1 immune score (IC)). The results indicate high reproducibility for each metric (R^2 range of 0.75–0.99).

Implementation of batch controls

Controls were included with each staining batch that were reviewed by a pathologist for approval. The controls included normal tonsil tissue and a tissue microarray with cores from a PD-L1$^+$ tumor and a PD-L1$^-$ tumor with high and low levels of all other markers in the panel. The pathologist compared the staining of the control sections within the current batch to that of the previous batches to approve or reject the quality control. If quality control was rejected, the issue was addressed and documented and all cases in the batch were re-stained.

Proximity score metric

The proximity score metric was formulated based on the G-cross function.[16,24,25] The G-cross function between cell types *A* and *B*, denoted as $G_{A,B}(r)$, represents the cumulative probability that a cell of type *B* is within a radius *r* from a cell of type *A*. We computed this function separately for each immune biomarker with respect to tumor cells for a given ROI. Explicitly, cell type *A* corresponded to cells positive for the tumor marker and cell type *B* corresponded to cells negative for the tumor marker and positive for a specific immune biomarker (CD8, FOXP3, PD-1, or PD-L1). The G-cross curve was then summarized into a single value using the area under the curve (AUC) up to a radius of 100 pixels (approximately 50 microns). This distance was selected to reflect relatively close proximity—on the order of several cell lengths—given that a typical cancer cell measures approximately 10–20 microns in diameter. The proximity score was then formulated as the ratio of this empirical AUC to a theoretical AUC that assumes random distribution of cells. Mathematically, the theoretical G-cross curve takes the form of $G_{A,B}(r) = 1 - e^{-\lambda_B \pi r^2}$ where λ_B is the density of cell type B in the ROI. Higher proximity scores thus indicate relatively closer immune cell-tumor cell proximity than would be expected based on the density of the immune cells alone. The proximity scores were calculated for ROIs for which at least 90% of cells fell in the segmented intratumor region and there were at least 20 cells of each type involved in the calculation. These criteria were employed to help mitigate potential confounders introduced by non-tumor regions and to standardize the calculations across cases, and because the relative proximity of cells is ill-defined/noisy with low cell counts. A case-level value was computed as the mean across ROIs. Both the density and proximity score metrics were performed at an individual biomarker level rather than combinations of biomarkers (e.g., CD8$^+$PD-1$^+$) because of the relatively low frequency of non-tumor cells positive for more than one biomarker, which would especially complicate the spatial analysis and hinder interpretability of the PCA. The R package *spatstat*[62] was used to compute the G-cross function.

Principal component analysis

The input variables to the PCA consisted of the cell density and proximity score for each immune biomarker (CD8, FOXP3, PD-1, PD-L1) and PD-L1 TPS. Each variable was normalized using a percentile ranking across the cohort to account for different scales across the variables and to emphasize the relative ranking across cases rather than the absolute magnitude. Missing proximity score values due to the cell count threshold described above were imputed using the median across the cohort. The rationale for this imputation is that the relative spatial arrangement of the immune cells within the ROI are neither closer nor farther away to the tumor than average, but rather undefined due to low counts, which is already accounted for in the cell density metric. The percentage of cases with imputed proximity scores was 15%, 30%, 28%, and 26% for CD8, FOXP3, PD-1, and PD-L1, respectively.

The PCA was performed across the entire cohort with subgroup analysis to assess consistency across specific case subsets (Figure S2). The number of PCs retained was chosen based on the value that was closest to explaining 90% of the variance, which resulted in five components (89% variance explained). To compare the PCs computed over different case subsets, a matching algorithm was first performed to map PC numbers across the subsets. Given two sets of PC weights, the absolute value in the correlation (*r*) between each pair of PCs between set 1 and set 2 was first computed. Starting with the first PC in set 1, the corresponding matching number in set 2 was assigned based on the highest correlation. This number was then removed from the list of possible matches and the process proceeded until a match was assigned for each PC in set 1. Figure S2 displays the resulting ordering and PC weights for different case subsets in comparison to the PCA fit across the cohort. Figure S3 displays the aggregate correlation in the PC weights between all pairs of subsets. The correlation is computed based on concatenating the weights of the first five PCs for each subset after the described alignment process.

Associations with cancer type and stage

The fraction of the variance in the TIME factor scores that is attributable to cancer type and stage was estimated using the variancePartition software,[27] which fits a linear mixed model. Cancer types with less than 20 cases were grouped as ‘Other’ for this analysis, and stage was binarized into low (0, I, II) or high (III, IV). Variations in the TIME factors for specific cancer types and stages were then visualized by binarizing the scores for each factor based on a median split across the cohort.

Associations with genomic alterations

1275 ImmunoProfile cases had available sequencing through OncoPanel (OP),[22,23] a custom hybrid-capture sequencing assay that targets exons of 304–447 genes across three panel versions (v1 $N = 22$ cases, v2 $N = 97$ cases, v3 $N = 1156$ cases). OncoPanel was performed separately from the ImmunoProfile assay as part of routine clinical genomics testing using either an alternative slice of the

same tissue block, or in some cases a different tissue block from the same specimen. TMB was calculated for the 1156 OPv3 cases as the number of coding somatic mutations that occur per megabase of exonic sequence data across all genes on the panel. Cancer type stratified van Elteren[63] tests were run to compare TIME spatial factor score distributions with genomic alterations using the *npsm*[64] package in R. For TMB, strata included cancer types that represented at least 10% of all cases with high TMB ($\geq$10 mutations/Mb), which included NSCLC, CRC, and melanoma with the remaining cancer types grouped as ‘Other’. Associations were assessed using the binarized high TMB status. For mutation and CNA gain/loss analyses, strata included cancer types that represented at least 5% of all alteration events for the alteration category, which consisted of the following; mutation events: bladder, CRC, endometrial, esophagogastric, melanoma, NSCLC, and pancreatic; CNA gains: breast, CRC, esophagogastric, melanoma, NSCLC, ovarian; CNA losses: breast, CRC, esophagogastric, melanoma, NSCLC, ovarian, pancreatic; with remaining cancer types grouped as ‘Other’ in each scenario. The list of mutations/CNA events included in the analysis was curated by starting with the genes present in OPv3, followed by annotation using OncoKB-Annotator (https://github.com/oncokb/oncokb-annotator) and filtering for alterations annotated as “oncogenic” or “likely oncogenic”.[29,30] To focus on pan-cancer effects, genes were further filtered to include only those with an alteration event in at least 5 different cancer types. Rare events (less than ten total alterations among all cases) were also excluded. For a given gene, all cases for which the gene is part of the OncoPanel version used for the case were included in the analysis (i.e., if a gene was in versions 1–3, all cases were included, whereas if it was only present in v3, only v3 cases were included). The full list of all tested genes, their panel version, and results are contained in Data S1, S2, and S3.

Associations with patient prognosis

Associations between the TIME spatial factors and patient prognosis were assessed using Cox proportional hazard (CoxPH) models[65] with cancer type and stage (low vs. high) as stratification variables. Models were fit separately for each TIME spatial factor using the entire cohort, for each cancer type with at least 80 cases, and for high and low stage. To explore multivariate prognostication, we calculated a composite TIME Score for each case by summing binarized values for each of the five TIME factors. Binarization was performed using a cohort-wide median split. For factors positively associated with survival (per univariate analysis), tumors above the median were assigned a value of 1, and those below, 0. For negatively associated factors, the assignment was reversed (1 for below-median, 0 for above-median). The resulting TIME Score ranged from 0 to 5.

QUANTIFICATION AND STATISTICAL ANALYSIS

For the genomics and prognosis analyses, the Benjamini-Hochberg method[28] was performed to correct for multiple comparisons with a false discovery rate of 10% for significance. All *p*-values are two sided unless otherwise noted. Statistical methods pertaining to specific analyses are described in their respective sections above.

Cell Reports Medicine, Volume *7*

Supplemental information

Pan-cancer spatial characterization of key immune biomarkers in the tumor microenvironment

James R. Lindsay, Jennifer Altreuter, Joao V. Alessi, Jason L. Weirather, Anita Giobbie-Hurder, Ian Dryg, Katharina Hoebel, Bijaya Sharma, Kristen Felt, F. Stephen Hodi, Neal Lindeman, Lynette M. Sholl, Ethan Cerami, Jonathan A. Nowak, Mark M. Awad, Scott J. Rodig, and William Lotter

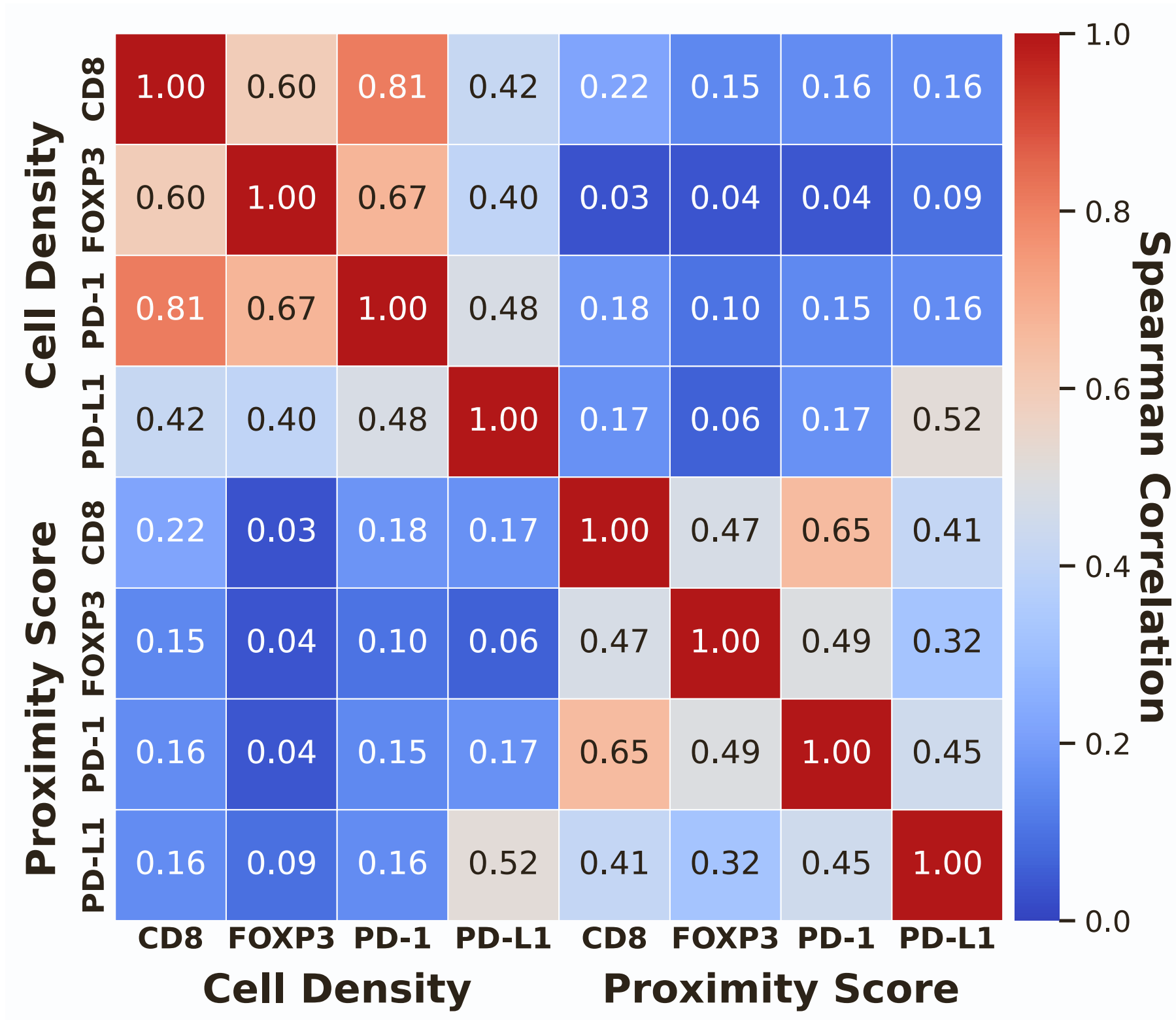

Figure S1. Correlations between the cell density and proximity score metrics across biomarkers. Related to Figure 2. Values represent the Spearman correlation computed across the cohort. Low correlations are generally observed between the cell density and proximity score metrics for the same biomarker, and moderate to high correlations are observed between different biomarkers for the same metric.

		Cell Density				Tumor	Proximity Score					
Factor	Subset	CD8	FOXP3	PD-L1	PD-1	TPS	CD8	FOXP3	PD-L1	PD-1	Variance	PC Rank
**Immune Enrichment**	All	0.41	0.36	0.44	0.42	0.39	0.22	0.16	0.27	0.21	43%	1
	NSCLC	0.38	0.34	0.43	0.38	0.35	0.27	0.17	0.32	0.28	40%	1
	BRCA	0.42	0.45	0.46	0.45	0.39	0.06	0.13	0.16	0.11	43%	1
	CRC	0.41	0.33	0.42	0.42	0.38	0.27	0.16	0.24	0.25	43%	1
	Other Cancer	0.41	0.35	0.44	0.43	0.39	0.21	0.15	0.27	0.20	43%	1
	Low Stage	0.41	0.37	0.44	0.42	0.40	0.18	0.14	0.27	0.19	43%	1
	High Stage	0.40	0.34	0.43	0.42	0.39	0.24	0.17	0.27	0.22	43%	1
	Genomics	0.40	0.35	0.43	0.41	0.38	0.26	0.17	0.27	0.22	43%	1
**Immune-Tumor Mixing**	All	-0.18	-0.28	-0.20	-0.22	-0.05	0.49	0.44	0.33	0.50	24%	2
	NSCLC	-0.25	-0.32	-0.19	-0.30	-0.05	0.47	0.45	0.25	0.47	22%	2
	BRCA	-0.10	-0.11	-0.10	-0.14	-0.01	0.57	0.51	0.35	0.49	23%	2
	CRC	-0.20	-0.31	-0.22	-0.22	-0.05	0.39	0.48	0.37	0.49	23%	2
	Other Cancer	-0.16	-0.26	-0.21	-0.20	-0.06	0.50	0.43	0.35	0.51	24%	2
	Low Stage	-0.15	-0.27	-0.18	-0.20	-0.06	0.51	0.44	0.33	0.51	23%	2
	High Stage	-0.19	-0.30	-0.21	-0.23	-0.06	0.47	0.44	0.32	0.50	24%	2
	Genomics	-0.21	-0.27	-0.22	-0.24	-0.06	0.46	0.45	0.33	0.50	23%	2
**Immune Evasion**	All	-0.33	-0.21	0.21	-0.28	0.54	-0.20	-0.28	0.54	-0.13	11%	3
	NSCLC	-0.33	-0.18	0.24	-0.32	0.55	-0.20	-0.30	0.50	-0.13	17%	3
	BRCA	-0.35	-0.19	0.14	-0.17	0.50	-0.21	-0.31	0.63	0.00	11%	3
	CRC	-0.21	-0.30	0.24	-0.20	0.56	-0.40	-0.07	0.50	-0.19	11%	3
	Other Cancer	-0.37	-0.22	0.22	-0.29	0.57	-0.20	-0.21	0.51	-0.15	11%	3
	Low Stage	-0.31	-0.23	0.19	-0.31	0.52	-0.18	-0.28	0.57	-0.14	11%	3
	High Stage	-0.34	-0.22	0.22	-0.27	0.55	-0.20	-0.27	0.53	-0.14	12%	3
	Genomics	-0.31	-0.22	0.21	-0.27	0.56	-0.22	-0.29	0.53	-0.12	12%	3
**Treg/Cytotoxic-Tumor Mixing**	All	-0.09	0.15	0.02	-0.09	0.07	-0.44	0.82	0.06	-0.31	6%	4
	NSCLC	-0.04	0.12	0.08	-0.08	0.06	-0.44	0.81	0.08	-0.33	6%	4
	BRCA	-0.12	0.12	-0.13	0.07	-0.09	-0.42	0.69	0.30	-0.45	4%	6
	CRC	0.09	0.25	-0.16	0.16	-0.23	-0.45	0.70	0.21	-0.30	7%	4
	Other Cancer	-0.16	0.31	-0.01	-0.10	0.02	-0.43	0.80	0.05	-0.22	7%	4
	Low Stage	-0.01	-0.04	0.03	-0.06	0.11	-0.40	0.83	0.03	-0.36	7%	4
	High Stage	-0.13	0.27	0.00	-0.11	0.03	-0.44	0.79	0.09	-0.26	6%	4
	Genomics	0.00	-0.01	0.05	-0.05	0.10	-0.40	0.82	0.04	-0.39	6%	4
**Treg/Cytotoxic Enrichment**	All	-0.43	0.78	-0.09	-0.17	-0.08	-0.12	-0.13	0.05	0.37	5%	5
	NSCLC	-0.48	0.81	-0.18	-0.14	0.00	-0.05	-0.07	-0.01	0.24	5%	5
	BRCA	-0.50	0.79	-0.27	-0.06	0.06	0.13	-0.13	-0.06	0.04	3%	7
	CRC	-0.59	0.65	0.10	-0.24	0.08	0.30	0.04	0.06	-0.21	5%	6
	Other Cancer	-0.38	0.75	-0.04	-0.23	-0.08	-0.01	-0.29	0.02	0.38	5%	5
	Low Stage	-0.51	0.71	0.02	-0.17	-0.03	-0.25	0.06	-0.02	0.38	5%	5
	High Stage	-0.39	0.79	-0.14	-0.17	-0.08	0.03	-0.25	0.10	0.30	5%	5
	Genomics	-0.38	0.83	-0.16	-0.16	-0.11	-0.20	0.01	0.18	0.19	5%	5

Figure S2. Results of PCA performed on different case subsets. Related to Figure 2. PC rank indicates the original order of the PC in terms of explained variance for the subset. High consistency is observed across the case subsets in terms of both the PC weights and variance explained. 'Other Cancer' includes cases from any cancer other than NSCLC, BRCA, and CRC. Genomics includes cases for which genomic profiling is available. NSCLC: non-small cell lung cancer, BRCA: breast cancer, CRC: colorectal cancer, PC: principal component, PCA: principal component analysis.

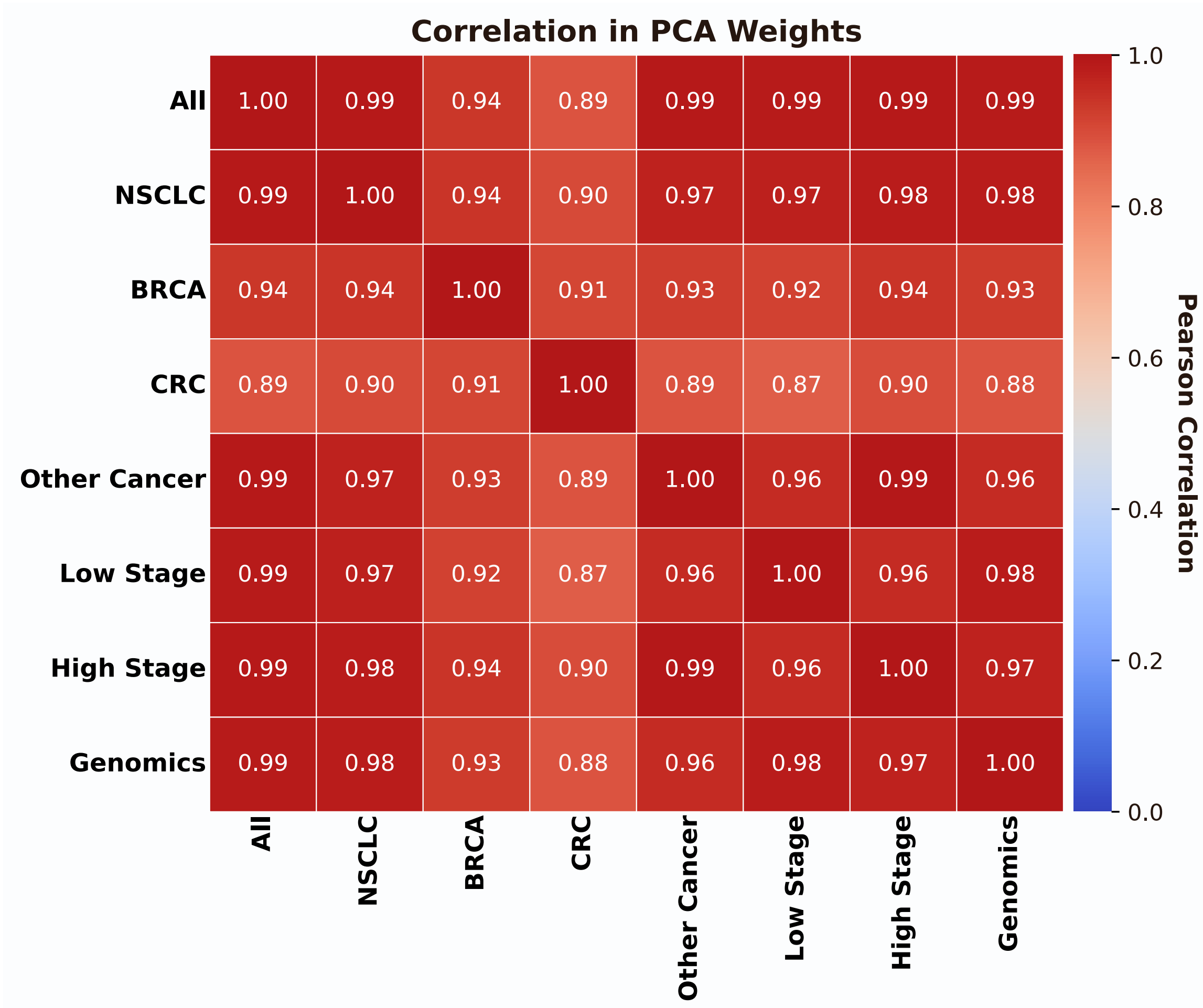

Figure S3. Correlation in principal component weights between different case subsets. Related to Figure 2. Correlation is computed between the five PCs representing the TIME spatial factors described in the text. High correlation is observed between each pair. 'Other Cancer' includes cases from any cancer other than NSCLC, BRCA, and CRC. Genomics includes cases for which genomic profiling is available. NSCLC: non-small cell lung cancer, BRCA: breast cancer, CRC: colorectal cancer, PC: principal component, PCA: principal component analysis.

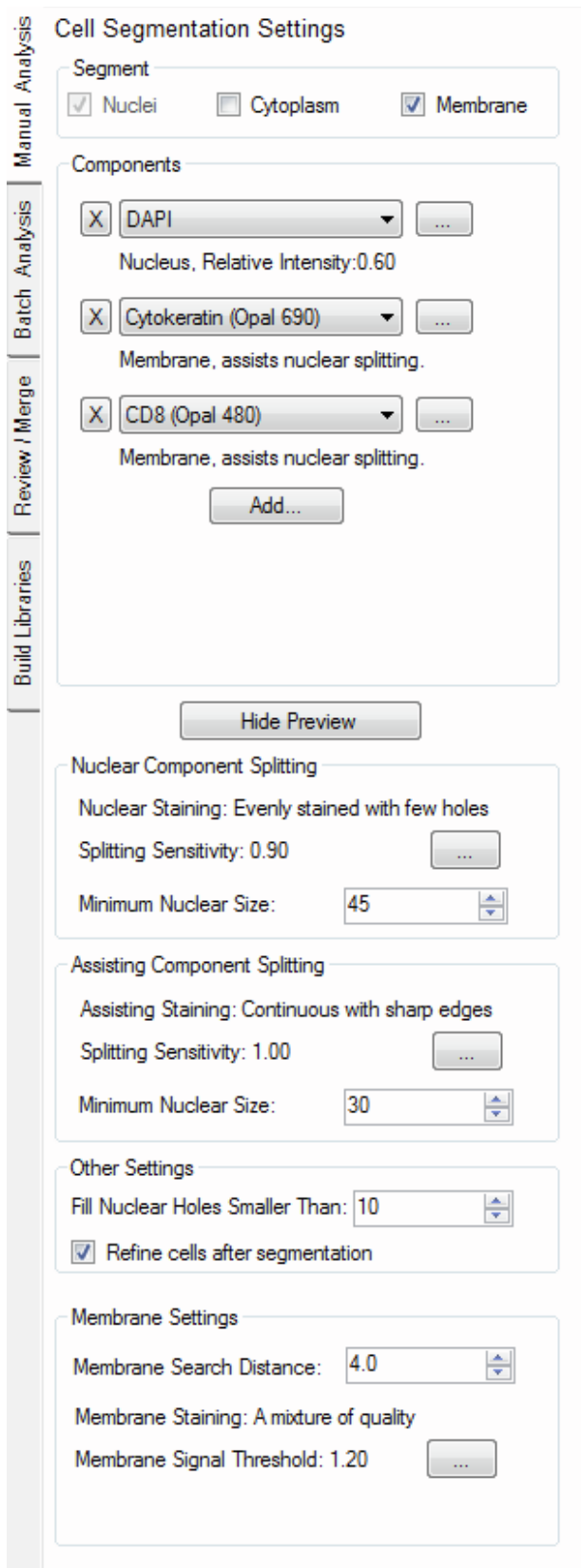

Figure S4. Default cell segmentation settings used in inForm. Related to STAR Methods. Cell segmentation was performed using a scientist-in-the-loop approach to refine the settings as necessary for a given case, followed by review and approval by a pathologist.

		Stage			Tissue Sample Type			Immunotherapy			Genomics	
**Cancer Type**	**Count (%)**	**Low**	**High**	**Unknown**	**Primary**	**Metastatic**	**Unknown**	**No**	**Yes**	**Unknown**	**No**	**Yes**
**Non-Small Cell Lung Cancer**	507 (25.1)	301 (59.4)	203 (40.0)	3 (0.6)	413 (81.5)	89 (17.6)	5 (1.0)	357 (70.4)	147 (29.0)	3 (0.6)	104 (20.5)	403 (79.5)
**Breast Cancer**	257 (12.7)	147 (57.2)	110 (42.8)	0 (0.0)	196 (76.3)	57 (22.2)	4 (1.6)	217 (84.4)	0 (0.0)	40 (15.6)	164 (63.8)	93 (36.2)
**Colorectal Cancer**	233 (11.5)	49 (21.0)	184 (79.0)	0 (0.0)	131 (56.2)	101 (43.3)	1 (0.4)	185 (79.4)	30 (12.9)	18 (7.7)	48 (20.6)	185 (79.4)
**Bladder Cancer**	160 (7.9)	101 (63.1)	59 (36.9)	0 (0.0)	139 (86.9)	21 (13.1)	0 (0.0)	107 (66.9)	53 (33.1)	0 (0.0)	89 (55.6)	71 (44.4)
**Esophagogastric Cancer**	123 (6.1)	35 (28.5)	84 (68.3)	4 (3.3)	91 (74.0)	31 (25.2)	1 (0.8)	83 (67.5)	20 (16.3)	20 (16.3)	46 (37.4)	77 (62.6)
**Melanoma**	108 (5.3)	23 (21.3)	84 (77.8)	1 (0.9)	42 (38.9)	65 (60.2)	1 (0.9)	10 (9.3)	98 (90.7)	0 (0.0)	31 (28.7)	77 (71.3)
**Pancreatic Cancer**	104 (5.2)	31 (29.8)	73 (70.2)	0 (0.0)	49 (47.1)	55 (52.9)	0 (0.0)	89 (85.6)	9 (8.7)	6 (5.8)	23 (22.1)	81 (77.9)
**Head and Neck Cancer**	95 (4.7)	38 (40.0)	55 (57.9)	2 (2.1)	76 (80.0)	19 (20.0)	0 (0.0)	53 (55.8)	40 (42.1)	2 (2.1)	61 (64.2)	34 (35.8)
**Endometrial Cancer**	94 (4.7)	55 (58.5)	38 (40.4)	1 (1.1)	77 (81.9)	17 (18.1)	0 (0.0)	75 (79.8)	4 (4.3)	15 (16.0)	49 (52.1)	45 (47.9)
**Ovarian Cancer**	92 (4.6)	18 (19.6)	73 (79.3)	1 (1.1)	42 (45.7)	50 (54.3)	0 (0.0)	78 (84.8)	3 (3.3)	11 (12.0)	30 (32.6)	62 (67.4)
**Renal Cell Carcinoma**	83 (4.1)	23 (27.7)	60 (72.3)	0 (0.0)	43 (51.8)	40 (48.2)	0 (0.0)	44 (53.0)	39 (47.0)	0 (0.0)	51 (61.4)	32 (38.6)
**Prostate Cancer**	35 (1.7)	8 (22.9)	27 (77.1)	0 (0.0)	19 (54.3)	16 (45.7)	0 (0.0)	27 (77.1)	8 (22.9)	0 (0.0)	12 (34.3)	23 (65.7)
**Hepatobiliary Cancer**	33 (1.6)	12 (36.4)	20 (60.6)	1 (3.0)	26 (78.8)	7 (21.2)	0 (0.0)	23 (69.7)	4 (12.1)	6 (18.2)	10 (30.3)	23 (69.7)
**Small Cell Lung Cancer**	20 (1.0)	5 (25.0)	15 (75.0)	0 (0.0)	12 (60.0)	7 (35.0)	1 (5.0)	13 (65.0)	7 (35.0)	0 (0.0)	10 (50.0)	10 (50.0)
**Other**	75 (3.7)	4 (5.3)	35 (46.7)	36 (48.0)	37 (49.3)	30 (40.0)	8 (10.7)	39 (52.0)	4 (5.3)	32 (42.7)	16 (21.3)	59 (78.7)
**Total**	2019 (100.0)	850 (42.1)	1120 (55.5)	49 (2.4)	1393 (69.0)	605 (30.0)	21 (1.0)	1400 (69.3)	466 (23.1)	153 (7.6)	744 (36.8)	1275 (63.2)

Table S1. Distribution of cases in cohort. Related to Figure 1. Tissue sample type indicates whether the specimen represents a primary or metastatic site. Genomics indicates the availability of genomic profiling. Immunotherapy indicates whether the patient was treated with immunotherapy either before or after the specimen date. Cancer types with less than 20 cases were grouped as 'Other' and include the following: Cancer of Unknown Primary (17 cases), Cervical Cancer (12), Thyroid Cancer (11), Anal Cancer (8), Gastrointestinal Neuroendocrine Tumor (5), Small Bowel Cancer (5), Salivary Gland Cancer (5), Non-Melanoma Skin Cancer (4), Mesothelioma (3), Ampullary Cancer (3), Vaginal Cancer (1), Thymic Carcinoma (1).

	Analysis Reproducibility (R^2)	
**Biomarker Metric**	**Same ROIs**	**Independent ROIs**
CD8 Density	0.96	0.90
FOXP3 Density	0.99	0.75
PD-1 Density	0.75	0.76
PD-L1 TPS	0.99	0.94
PD-L1 IC	0.86	0.80

Table S2. Image Analysis Reproducibility Results. Related to STAR Methods. Two scientists independently performed the image analysis for a set of 20 cases, once using the same ROIs and once with independently selected ROIs. TPS: tumor proportion score; IC: immune score (% of intratumor area occupied by PD-L1+ immune cells).
